# Unraveling the role of ADAMs in clinical heterogeneity and the immune microenvironment of hepatocellular carcinoma: insights from single-cell, spatial transcriptomics, and bulk RNA sequencing

**DOI:** 10.3389/fimmu.2024.1461424

**Published:** 2024-09-13

**Authors:** Junhong Chen, Qihang Yuan, Hewen Guan, Yuying Cui, Chang Fu, Tianfu Wei, Kai Liu

**Affiliations:** ^1^ Department of Hepatobiliary and Pancreatic Surgery, General Surgery Center, The First Hospital of Jilin University, Changchun, China; ^2^ Department of Oncology, The First Affiliated Hospital of Dalian Medical University, Dalian, China; ^3^ Clinical Laboratory of Integrative Medicine, First Affiliated Hospital of Dalian Medical University, Dalian, China

**Keywords:** hepatocellular carcinoma, ADAM family, immune microenvironment, single-cell sequencing, spatial transcriptome sequencing, bulk RNA sequencing, clinical heterogeneity

## Abstract

**Background:**

Hepatocellular carcinoma (HCC) is a prevalent and heterogeneous tumor with limited treatment options and unfavorable prognosis. The crucial role of a disintegrin and metalloprotease (ADAM) gene family in the tumor microenvironment of HCC remains unclear.

**Methods:**

This study employed a novel multi-omics integration strategy to investigate the potential roles of ADAM family signals in HCC. A series of single-cell and spatial omics algorithms were utilized to uncover the molecular characteristics of ADAM family genes within HCC. The GSVA package was utilized to compute the scores for ADAM family signals, subsequently stratified into three categories: high, medium, and low ADAM signal levels through unsupervised clustering. Furthermore, we developed and rigorously validated an innovative and robust clinical prognosis assessment model by employing 99 mainstream machine learning algorithms in conjunction with co-expression feature spectra of ADAM family genes. To validate our findings, we conducted PCR and IHC experiments to confirm differential expression patterns within the ADAM family genes.

**Results:**

Gene signals from the ADAM family were notably abundant in endothelial cells, liver cells, and monocyte macrophages. Single-cell sequencing and spatial transcriptomics analyses have both revealed the molecular heterogeneity of the ADAM gene family, further emphasizing its significant impact on the development and progression of HCC. In HCC tissues, the expression levels of ADAM9, ADAM10, ADAM15, and ADAM17 were markedly elevated. Elevated ADAM family signal scores were linked to adverse clinical outcomes and disruptions in the immune microenvironment and metabolic reprogramming. An ADAM prognosis signal, developed through the utilization of 99 machine learning algorithms, could accurately forecast the survival duration of HCC, achieving an AUC value of approximately 0.9.

**Conclusions:**

This study represented the inaugural report on the deleterious impact and prognostic significance of ADAM family signals within the tumor microenvironment of HCC.

## Introduction

Hepatocellular carcinoma (HCC) is a common and heterogeneous tumor with increasing incidence worldwide (1, 2). It is associated with various risk factors, and although there are treatment options available, the prognosis for patients remains unfavorable (3, 4). In China, HCC is particularly prevalent and is the second most common malignancy, with most patients being diagnosed in intermediate or advanced stage (5). And as a result, the patients miss out on the chance for radical surgery. Although surgical resection, percutaneous approaches and liver transplantation are potential treatment options, postoperative recurrence and metastasis pose significant challenges (6). For the past few years, targeted therapy and immune checkpoint inhibitors have shown promise but also have limited efficacy for some patients (7, 8). Therefore, novel and reliable screening methods are urgently needed to improve diagnosis and treatment, and new targets must be explored to enhance therapeutic efficacy for HCC patients.

A disintegrin and metalloprotease (ADAM) family genes are a group of genes that encode proteases that can affect many physiological and pathological processes, including the occurrence and development of tumors (9). There are about 30 members of the ADAM family, whose functions and expressions vary in different types of tumors. They have a distinct structure that includes adhesion and protease domains. The specific ADAM proteins encoded by these genes are devoted in the occurrence and development of tumors by modulating the TNF-alpha, E-cadherin, receptor-II, CD44, L-selectin, Notch, Erb4/HER4 and so on. All of these proteins play important roles in cancer cell proliferation, migration and invasion. An ADAM member known as ADAM10 has been specifically identified as an oncogene that contributes to the progression of HCC. Knocking down ADAM10 has been shown to significantly inhibit the proliferation, migration, and invasion of HepG2 cells, highlighting its critical role in HCC development (10). In addition, the ADAM10 can impair the recognition of cancer cells by T or NK cells by shedding the “stress-induced” molecules MICA, MICB, and ULBPs expressed on the cancer cell surface, which are responsible for inducing an immune response against cancer cells upon binding to NKG2D receptors expressed on natural killer (NK) cells and most cytotoxic T lymphocytes (11–14). In addition to the ADAM10 gene, ADAM9 and ADAM17 are the other frequently reported genes in liver cancer, playing a crucial regulatory role in chemotherapy resistance and malignant tumor progression (15, 16). The small molecule drug CCL347, which targets ADAM9, can enhance the tumor-killing ability of NK cells, making it a promising new treatment for HCC (17). The small molecule inhibitor ZLDI-8, which targets ADAM17, can inhibit HCC metastasis and enhance the therapeutic sensitivity to sorafenib (18, 19). In summary, most previous studies have focused on the mechanistic research and drug development of individual ADAM gene. There is still a lack of a comprehensive understanding of the role of the ADAM gene family in HCC. Additionally, with the rapid advancement of omics technologies, their multi-omics characteristics remain to be explored.

In our research, the fusion of single-cell sequencing and spatial transcriptomics technologies has, for the first time, offered a comprehensive glimpse into the single-cell states and spatial distribution of ADAM family genes. This enabled the simulation of ADAM family signal intensity. Patients with HCC were subsequently categorized into three groups: high ADAM, medium ADAM, and low ADAM signal levels, facilitating a comparison of prognosis and the tumor microenvironment across these groups. PCR and IHC validation was employed to confirm the abnormal expression patterns of ADAM family genes in HCC. After evaluating 99 traditional machine learning algorithms, a novel prognostic model was established. In essence, this study has illuminated the molecular attributes of ADAM in HCC, offering a comprehensive perspective through single-cell and spatial genomics, and introducing a precise prognostic model for the first time.

## Methods

### Single-cell sequencing analysis of ADAM family genes in HCC

In March 2023, we collected cancer tissue and adjacent tissue samples from three pairs of hepatocellular carcinoma for single-cell sequencing. These samples were all obtained from the First Hospital of Jilin University(ID: AF-IRB-032-06). All patients provided informed consent for donating their samples for scientific research before surgery, and the study was approved by the ethics committee of the First Hospital of Jilin University. Additionally, we also collected two publicly available single-cell sequencing samples of hepatocellular carcinoma for validation, namely GSM5076749 and GSM5076750 (20).

The single-cell sequencing process based on 10x Genomics proceeds as follows: Using the Chromium™ Single Cell 3’Solution microfluidic platform, gel beads containing barcodes and primers are encapsulated with individual cells in droplets, forming Gel Bead in Emulsion (GEM). Prepared cell suspension, 10X barcode gel beads, and droplets are separately introduced into different channels of Chromium Chip B, creating a single-cell reaction system through the microfluidic “double-cross” system. The gel beads inside the GEM dissolve, leading to cell lysis and mRNA release, followed by reverse transcription to generate barcoded cDNA for sequencing. After breaking the liquid oil layer, cDNA amplification is performed, followed by purification, quality control, library construction, and library sequencing once it passes the quality check.

We applied consistent quality control and analysis strategies to both self-generated data and publicly available data. The specific process is as follows: Leveraging the provided 10x Genomics sequencing data files, which include barcodes, features, and matrix files, we imported the single-cell data through the utilization of the Read10X and CreateSeuratObject functions within the R package Seurat. We established specific criteria for data filtration, which encompassed the following conditions: min.cells = 3, min.features = 200, nCount_RNA >= 1000, nFeature_RNA >= 200, nFeature_RNA <= 10000, percent.mt <= 20, percent.rb <= 20.

We utilized the VlnPlot function to create violin plots that visually represented the following metrics: “nFeature_RNA,” “nCount_RNA,” “percent.mt,” and “percent.rb.” Additionally, we employed the FeatureScatter function to assess the correlations between these metrics. For data standardization, we applied the NormalizeData function with the normalization method set to “LogNormalize”. Furthermore, the FindVariableFeatures function was used to identify the top 2000 highly variable genes for subsequent principal component analysis (PCA) dimensionality reduction. Before conducting PCA dimensionality reduction, we performed data normalization using the ScaleData function, a crucial preprocessing step. Subsequently, we executed PCA dimensionality reduction. To mitigate potential batch effects stemming from different sample sources, we applied the well-established RunHarmony function to harmonize the single-cell data. The determination of the number of principal components to use was based on results obtained from the JackStraw and ScoreJackStraw functions. To establish neighborhood relationships and evaluate clustering outcomes at various resolutions, we made use of the FindNeighbors function. Additionally, we employed the clustree function to pinpoint the optimal resolution parameter while taking measures to mitigate batch effects between samples.

Following the completion of dimensionality reduction and clustering at the single-cell level, we employed the FindAllMarkers function to pinpoint specific expression markers for each cell subtype. These identified markers were subsequently utilized for cell annotation analysis. In this research, we harnessed the well-established SingleR function for automated cell subtype annotation. SingleR’s operational principle is grounded in the similarity of gene expression patterns. It leverages gene expression data from established reference datasets containing information about cell types or phenotypes to predict the cell type of individual cells. This approach proves exceptionally valuable for the identification and categorization of distinct cell populations within single-cell RNA sequencing data, providing researchers with insights into the distribution and characteristics of various cell types within their samples. The reference dataset employed in this study is defined as follows: refdata = celldex::HumanPrimaryCellAtlasData(). In addition, copykat algorithm was utilized to predict the benign and malignant nature of each cell.

We conducted a comprehensive evaluation of ADAM family signal scores for each cell using six well-established single-cell gene set scoring algorithms: Add, AUCell, UCell, singscore, ssgsea, and total scoring (21–23). The specific calculation process is as follows: 1) The AddModuleScore function was employed to assist in the assessment of ADAM family signal scores under the Add mode. 2) The AUCell_buildRankings and AUCell_calcAUC functions were utilized to compute ADAM family signal scores under the AUCell mode. 3) The irGSEA.score function was applied to calculate ADAM family signal scores under both the UCell and singscore modes. 4) The gsva function was utilized to evaluate ADAM family signal scores under the ssgsea mode. 5) Finally, the results obtained from the five calculations mentioned above were summed to derive the total scoring value.

### Spatial transcriptome sequencing analysis of ADAM family genes in HCC

The spatial transcriptomic data for HCC patients were sourced from the GSE238264 dataset, which encompasses spatial transcriptomic data from 7 eligible HCC cases specifically denoted as GSM7661255, GSM7661256, GSM7661257, GSM7661258, GSM7661259, GSM7661260, and GSM7661261 (24). We employed the Read10X function to ingest cell expression data from the spatial transcriptomics, and the Read10X_Image function was utilized to extract spatial cell location information.

In parallel with the preceding methodologies employed in single-cell sequencing analysis, we conducted a comprehensive assessment of ADAM signals within the spatial context of HCC utilizing six established gene set scoring algorithms. These six algorithms encompass Add, AUCell, UCell, singscore, ssgsea, and total scoring. The specific calculation process closely paralleled the previously outlined procedures (22).

### Pan-cancer analysis overview of the ADAM family genes

Following a methodology akin to previous studies (25–27), we compiled and meticulously curated comprehensive pan-cancer genetic and transcriptomic datasets from the TCGA database. These datasets encompassed SNV (Single Nucleotide Variation) data, CNV (Copy Number Variation) data, methylation data, and RNA sequencing data. The SNV data were sourced from the following website: https://gdc.cancer.gov/about-data/publications/pancanatlas. Our SNV analysis was executed using the maftools package, and the results were visually presented using the oncoplot and ggplot functions.

For CNV analysis, we obtained data from the XENA website using the dataset ID: TCGA.PANCAN.sampleMap/Gistic2_CopyNumber_Gistic2_all_data_by_genes. We set thresholds for CNV amplification and deletion at 5%, and similarly, the visualization of CNV results was accomplished using the ggplot function.

The visualization analysis of methylation data was also conducted utilizing the ggplot function. In the methylation heatmap, particular attention was paid to the size and color of the dots, with larger dots signifying greater statistical significance. Yellowish dots indicated a proclivity toward higher methylation levels, while bluish dots suggested a tendency toward lower methylation levels.

Differential expression analysis was undertaken through the utilization of the limma package. The expression heatmap highlighted both the size and color of the dots, with larger dots denoting higher statistical significance. Reddish dots indicated an inclination toward upregulation, whereas greenish dots signified a tendency toward downregulation of genes.

### Bulk RNA sequencing analysis of ADAM family genes in HCC

Molecular clustering analysis was conducted using the transcriptomic data from the TCGA-HCC dataset. This dataset comprises 343 HCC samples with well-defined prognostic information and 50 matched adjacent non-cancerous tissue samples. Due to significant variations in ADAM gene expression and variation levels identified in the pan-cancer analysis, we developed a classification model to account for these disparities in ADAM expression levels among different HCC samples. Our approach involved employing ssGSEA analysis to assess ADAM family scores for each patient. To delve into the variance, we utilized the “gplots” package in R and generated heatmaps based on cluster analysis results using the “pheatmap” package (28). We then categorized all samples into three distinct clusters based on the mRNA expression levels of ADAM-related genes, classifying them as ADAM-high, ADAM-medium, or ADAM-low. To effectively visualize the correlation between gene expression levels within these three clusters, we turned to the “ggpubr” package, which allowed us to create a violin plot showcasing the enrichment scores for each cluster. Lastly, we delved into disparities in patient outcomes among these three clusters using the “survival” package and the “surminer” package within R Studio (29).

To delve into the tumor microenvironment within the three clusters, we employed a range of algorithms, including TIMER, QUANTISEQ, MCPCOUNTER, XCELL, EPIC, and CIBERSORT, for further analysis. Furthermore, we compared the expression levels of common immune checkpoint genes across the three clusters using the Kruskal-Wallis test. Using ssGSEA, we estimated the immune response and proceeded to explore the correlations between ADAM family gene expression and the infiltration of immune cells, visualizing the results in a heatmap. Additionally, we investigated the correlations between ADAM scores and the infiltration of immune cells.

In addition to the TCGA-HCC dataset, we also gathered data from the GSE116174 (30), GSE144269 (31), GSE76427 (32), and Meta datasets to develop a robust prognosis model based on ADAM co-expression networks. The Meta dataset is a compilation of HCC samples from the first four datasets, and its significance lies in providing a more objective assessment of the authenticity and applicability of our developed model. Similar to previous research methodologies, for transcriptomic data originating from different dataset sources, we also employed the sva package to mitigate batch effects as comprehensively as possible. Additionally, we performed LOG transformation based on the data’s range of variation. Our approach began with identifying complex networks of co-expressed genes among the ADAM family genes using Spearman’s test. The genes that were identified as having prognostic values in at least four cohorts through univariate Cox regression analysis were retained for subsequent model construction. Subsequently, the genes within these networks were curated and employed in the subsequent model construction analysis.

In order to create a robust consensus model that achieves high accuracy and stability, we combined 10 machine learning algorithms (i.e. RSF, Enet, Lasso, Ridge, stepwise Cox, CoxBoost, plsRcox, SuperPC, GBM, survival-SVM) and explored 99 different algorithm combinations. The signature generation procedure was as follows: (a) Univariate Cox regression analysis was conducted to identify variables associated with HCC prognosis; (b) Variables that demonstrated prognostic value in at least four HCC datasets were retained for modeling;(c) We conducted 99 algorithm combinations on the prognostic features to develop prediction models using the leave-one-out cross-validation framework within the TCGA-HCC cohort; (d) Patients in four validation datasets (GSE116174, GSE144269, GSE76247, and the Meta dataset) were categorized into high-score and low-score groups according to the median value. KM survival analysis and ROC curves were carried out to validate the predictive performance of our model.

Considering the limited number of liver cancer samples in the TCGA cohort, we further validated the expression level of ADAM10 using additional liver cancer transcriptome datasets. The BEST website (https://rookieutopia.com/app_direct/BEST/#PageHomeAnalysisModuleSelection), which provides a curated database and innovative analytical pipelines for exploring cancer biomarkers at high resolution, was utilized in our study. Subsequently, we analyzed the transcriptomic features of ADAM10 in E_TABM_36, GSE144269, GSE14520, and GSE54236 datasets on the BEST platform. Furthermore, the BEST platform also provided comprehensive clinical information of multiple liver cancer cohorts, including TCGA, E_TABM_36, GSE144269, GSE14520, GSE10141, GSE104580, and GSE109211. Therefore, we investigated the tight correlation between the expression level of ADAM10 and various clinical parameters of liver cancer, including HBV status, liver cirrhosis status, tissue grading, AFP level, satellite lesion, transcatheter arterial chemoembolization, and Sorafenib treatment sensitivity.

### Cell culture and real-time quantitative polymerase chain reaction

Firstly, we predicted the expression patterns of ADAM family genes between HCC tumor tissues and adjacent non-cancerous tissues using the GEPIA2 platform (33). Subsequently, we validated the differentially expressed ADAM family genes through three HCC cell lines (HepG2, HuH7, and Hep3B2.1-7) and one normal liver cell line (L-02). HuH7 cells were cultured in DMEM high glucose medium, HepG2 and Hep3B2.1-7 cells were cultured in MEM medium, and the L-02 cell line was cultured in RPMI-1640 medium. All culture media were supplemented with 10% fetal bovine serum and 1% penicillin-streptomycin solution. Cells were maintained in a cell incubator at 37°C with a 5% CO2 atmosphere.

In the four cell lines, total RNA was extracted using the conventional Trizol method, and cDNA was synthesized using a reverse transcription kit. Subsequently, we assessed the expression levels of the target gene using a SYBR Green I fluorescent dye-based assay, with β-actin serving as the internal reference gene. The RNA levels were analyzed and quantified utilizing the 2^-ΔΔCt method.

### HCC tissue microarray and immunohistochemistry experiments

We purchased human HCC TMA from Zhuoli Biotechnology Co. in Shanghai, China. The tissue sections were initially subjected to antigen retrieval using EDTA after dewaxing. Subsequently, the sections were treated with primary antibodies, followed by incubation with secondary antibodies. Diaminobenzidine staining was then performed, and hematoxylin was used for counterstaining. This process allowed us to complete the immunohistochemistry (IHC) assessment for 48 HCC samples and 48 normal samples. Two independent pathologists assessed the H-scores of ADAM10 protein, taking into account the staining intensity and the percentage of positively stained cells.

## Results

### Our self-sequencing single-cell data for ADAM family genes in HCC

**Figure 1:**
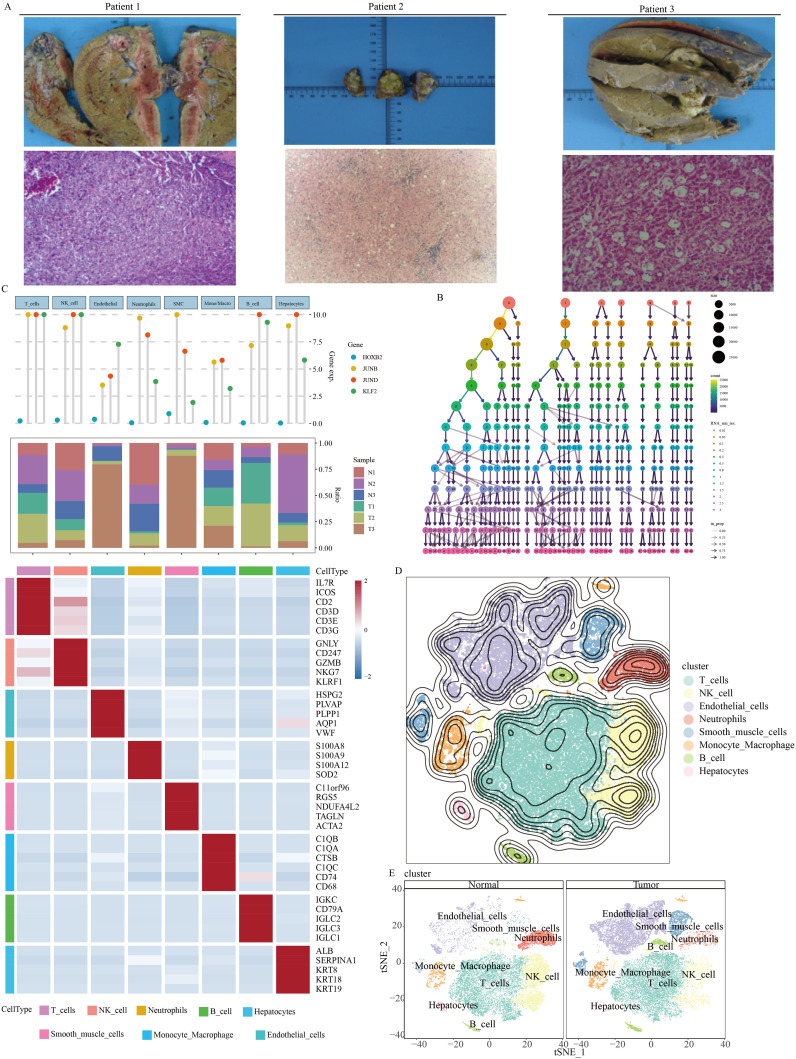
Single-cell overview of HCC in our self-generated cohort. **(A)** Pathology imaging of HCC patients **(B)** Determination of appropriate resolution. **(C)** Cell type annotation. **(D)** Overview of t-SNE dimensionality reduction. **(E)** t-SNE dimensionality reduction features of normal tissue and tumor tissue.

The pathology imaging of 3 paired HCC patients were summarized in **Figure 1A**. Our self-sequencing HCC single-cell data encompassed a total of 63,998 cells. Following the application of filters using criteria such as nCount_RNA, nFeature_RNA, percent.mt, and percent.rb metrics, 47299 cells met the criteria and were retained for further single-cell analysis. The quality control results of the single-cell data were depicted in **Supplementary Figures 1A, 1B**. The data processing steps using the Harmony algorithm were illustrated in **Supplementary Figures 1C, 1D**. **Supplementary Figure 1C** represents the clustering plot before batch correction with Harmony, while **Supplementary Figure 1D** shows the clustering plot after batch correction using Harmony.

According to **Figure 1B**, it was evident that setting the resolution to 1.5 might yield an optimal clustering result. Results from the SingleR automatic annotation indicated the presence of primarily 8 cell types within the HCC microenvironment, including T cell, NK cell, neutrophils, B cell, hepatocyte, smooth muscle cell, monocyte-macrophage, and endothelial cells (**Figures 1C, D**).

**Figure 2:**
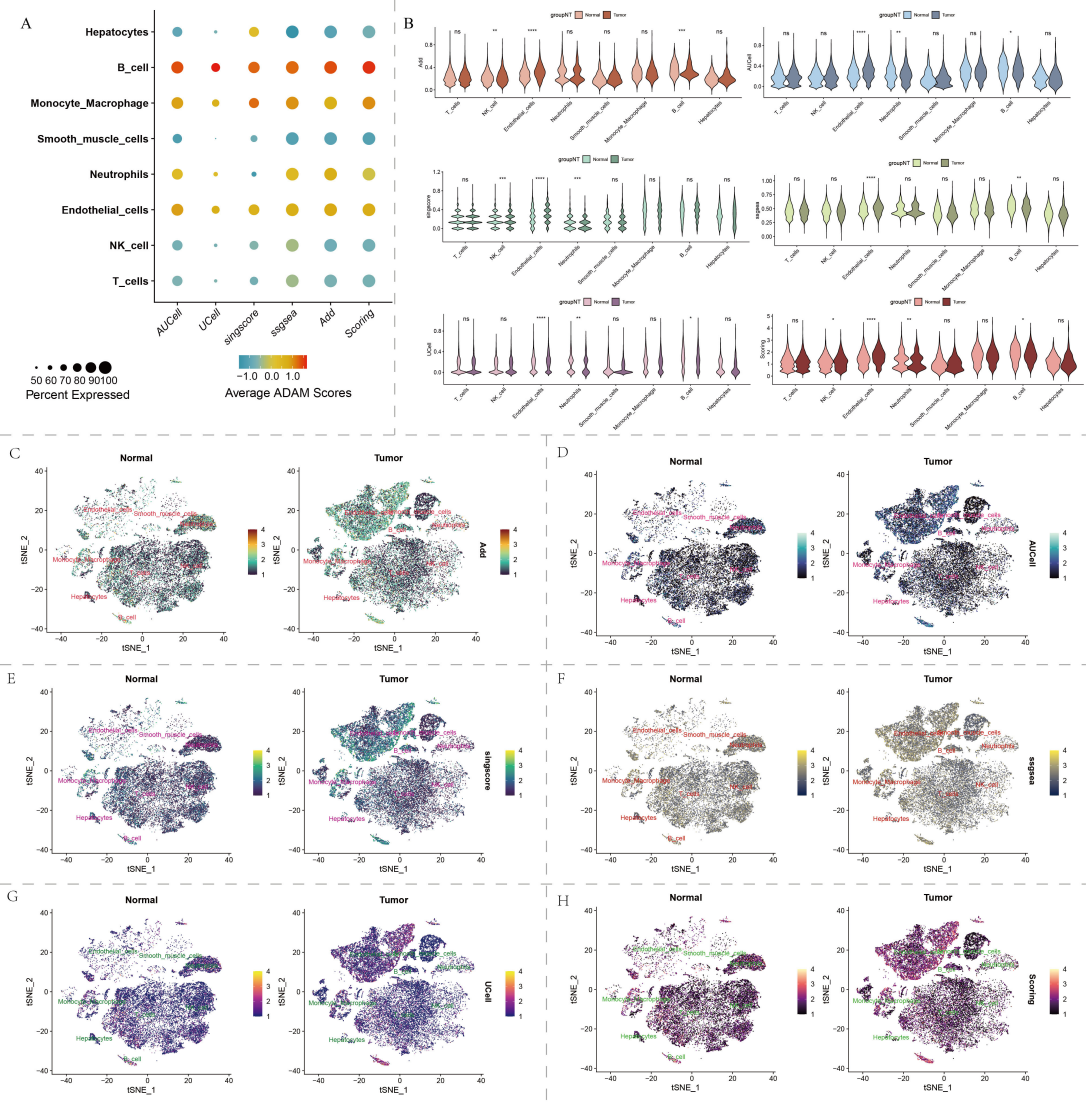
Single-cell distribution of ADAM family signals in our self-generated cohort. **(A)** Bubble chart displays the ADAM signals for each type of cell. **(B)** Violin plot displays the ADAM signals based on six algorithms. **(C–H)** t-SNE dimensionality reduction displays the single-cell distribution of ADAM signals. Six algorithms used for assessing ADAM signals involve AUCell, UCell, Add, singscore, ssgsea, and Scoring. (*:p<0.05,**:p<0.01,***:p<0.001,****:p<0.0001; p value was calculated by wilcox.test).

The cellular composition of HCC tumor tissue differed significantly from its adjacent non-cancerous tissue (**Figure 1E**). B cells and endothelial cells exhibited notably active ADAM signals (**Figure 2A**). Importantly, various cell types within the tumor tissue displayed abnormal ADAM signals when compared to the non-cancerous tissue, suggesting a potential role of ADAM signaling in the process of HCC development (**Figure 2B**). To visualize the ADAM signal of each cell more clearly, we subsequently projected the ADAM signal scores onto the previous t-SNE clustering plot. The results indicated that endothelial cells within the tumor tissue were the primary source of abnormally active ADAM signals (**Figures 2C-H**).

**Figure 3:**
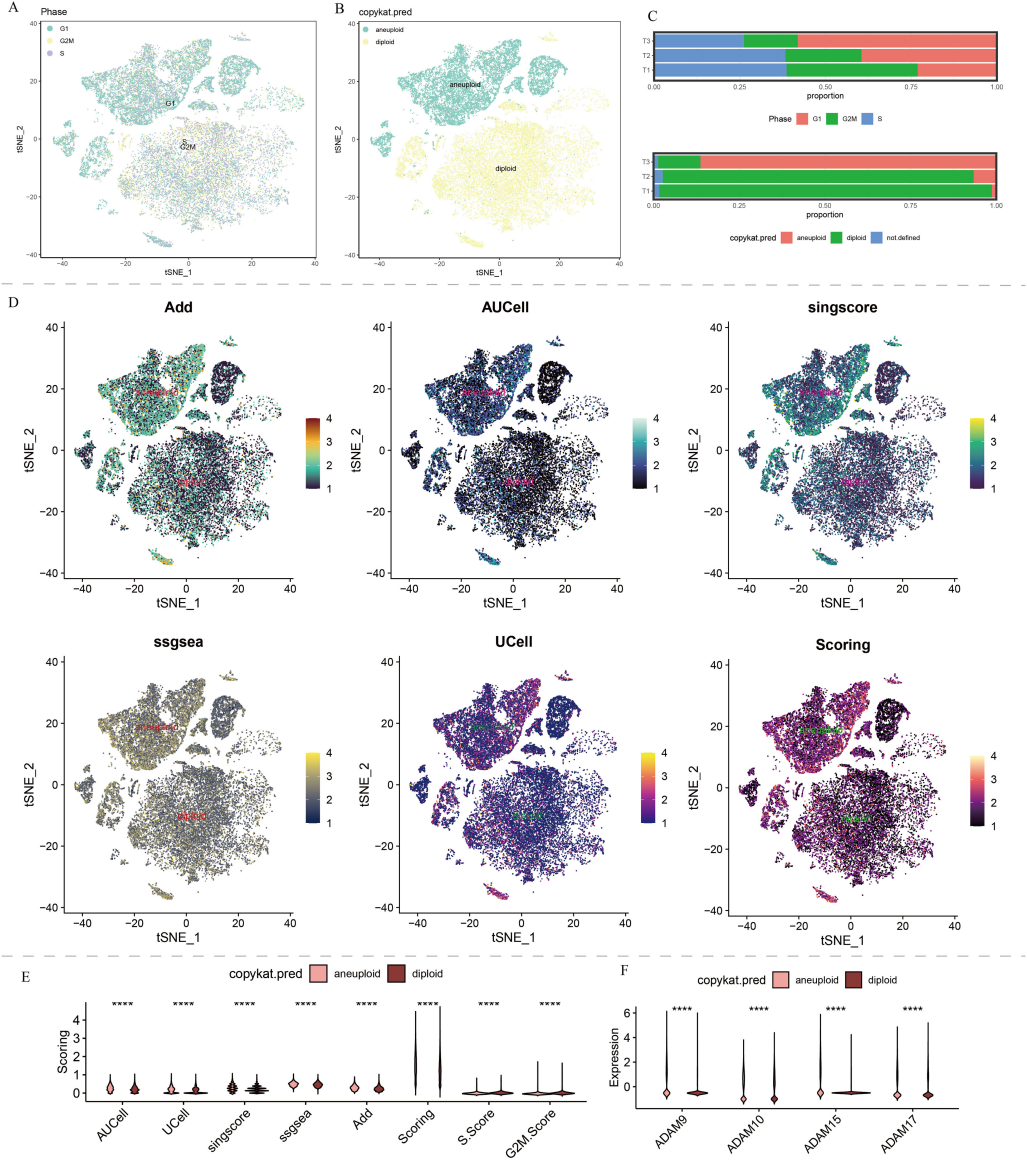
Copykat results and cell cycle analysis of single-cell data in our self-generated cohort. **(A)** t-SNE dimensionality reduction of copykat results. **(B)** t-SNE dimensionality reduction of cell cycle analysis. **(C)** The proportion of G1, S, and G2M in HCC, and proportion of aneuploid and diploid in HCC. **(D)** Single-cell distribution of ADAM signals in aneuploid and diploid. **(E)** The violin plot displays the discrepancies in ADAM signals between aneuploid and diploid. **(F)** The violin plot displays the discrepancies in expression of ADAM9, ADAM10, ADAM15, and ADAM17 between aneuploid and diploid. (****:p<0.0001; p value was calculated by wilcox.test).

Furthermore, we found that endothelial cells, monocyte-derived macrophages, and smooth muscle cells primarily resided in the G1 phase, while other cell types showed a more even distribution in the G1/S/G2M phases (**Figure 3A**). Hepatocytes, endothelial cells, and smooth muscle cells were the major constituents of malignant cells in HCC patients (**Figure 3B**). The cell cycle distribution and malignancy assessment results for each tumor sample were depicted in **Figure 3C**. Once again, the results consistently demonstrated that malignant cells exhibited more active ADAM signals, especially in endothelial cells and hepatocytes, compared to benign cells (**Figure 3D**).

Regardless of the predictive algorithm used, there was a clear statistical difference in ADAM signals between benign and malignant cells, with malignant cells exhibiting higher G1 phase signals and benign cells showing higher S phase and G2M phase signals (**Figure 3E**).

### Public single-cell sequencing and spatial transcriptome sequencing analysis for ADAM family genes in HCC

The public single-cell sequencing results of HCC yielded a total of 10,729 qualified cells remained for subsequent single-cell analysis (**Supplementary Figures 2A, B**). To mitigate batch effects in the samples, harmony analysis was applied to modify the single-cell data to some extent. The cell distribution results before and after harmony analysis were shown in **Supplementary Figures 2C, 2D**, indicating that harmony analysis partially reduced the batch effects in the samples, leading to a more uniform cell distribution among the samples.

Highly variable gene selection and Elbowplot results indicated that pc=10 was the clear inflection point, where most of the true signals are captured by the first 10 principal components (**Supplementary Figure 2E**). Under a resolution of 1.5, the 10,729 qualified cells were divided into 22 clusters (**Supplementary Figure 2F**). High-variance genes were identified for each cluster, and cell annotation analysis was conducted, resulting in the identification of 6 cell types: monocytes/macrophages (CD68, CD74, C1QA, C1QB, C1QC, SPP1), T cells (CD2, CD3D, CD3E, CD3G, NKG7, GZMA), B cells (CD79A, IGLC2, IGLC3), hepatocytes (KRT18, KRT19, SPINK1, TSPAN8), smooth muscle cells (RGS5, NDUFA4L2, TAGLN, ACTA2), and endothelial cells (HSPG2, PLVAP, STC1, AQP1, VMF) (**Supplementary Figure 3A**). Transcription factors JUNB and JUND exhibit higher expression levels in monocytes/macrophages, T cells, smooth muscle cells, and endothelial cells (**Supplementary Figure 3A**). The t-SNE and UMAP dimensionality reduction results for these 6 cell types were shown in **Supplementary Figures 3B, 3C**, indicating that each cell type was well-separated from others, demonstrating a good annotation outcome.

Using the AUCell, UCell, Add, singscore, ssgsea, and scoring algorithms, we predicted the distribution of the ADAM family signals at single-cell resolution across different cell types (**Supplementary Figure 4A**). The strongest ADAM signals were observed in endothelial cells and monocytes/macrophages, followed by hepatocytes, while the weakest ADAM signals were found in T cells, B cells, and smooth muscle cells (**Supplementary Figure 4B**). The t-SNE plot further confirmed that the ADAM signals in subpopulations of endothelial cells and monocytes/macrophages were much stronger compared to other cell types (**Supplementary Figure 4C**).

The copykat results indicated that nearly all liver cells in these two HCC patients were malignant cells, while immune cells and smooth muscle cells were benign cells. This aligns with our common understanding, demonstrating the accuracy of copykat’s predictions (**Supplementary Figure 5A**). Cell cycle analysis results showed that malignant liver cells consisted of cells in the G1, S, and G2M phases, with a predominant population in the G1 phase (**Supplementary Figure 5B**). In contrast, smooth muscle cells, endothelial cells, T cells, and B cells were primarily in the G1 phase, with rare occurrences of cells in the G2M and S phases (**Supplementary Figure 5B**). The distribution of cells in the G1, S, and G2M phases was relatively uniform within the two HCC samples (**Supplementary Figure 5C**). The copykat predictions for samples GSM5076749 and GSM5076750 were depicted in **Supplementary Figure 5D**.

**Figure 4:**
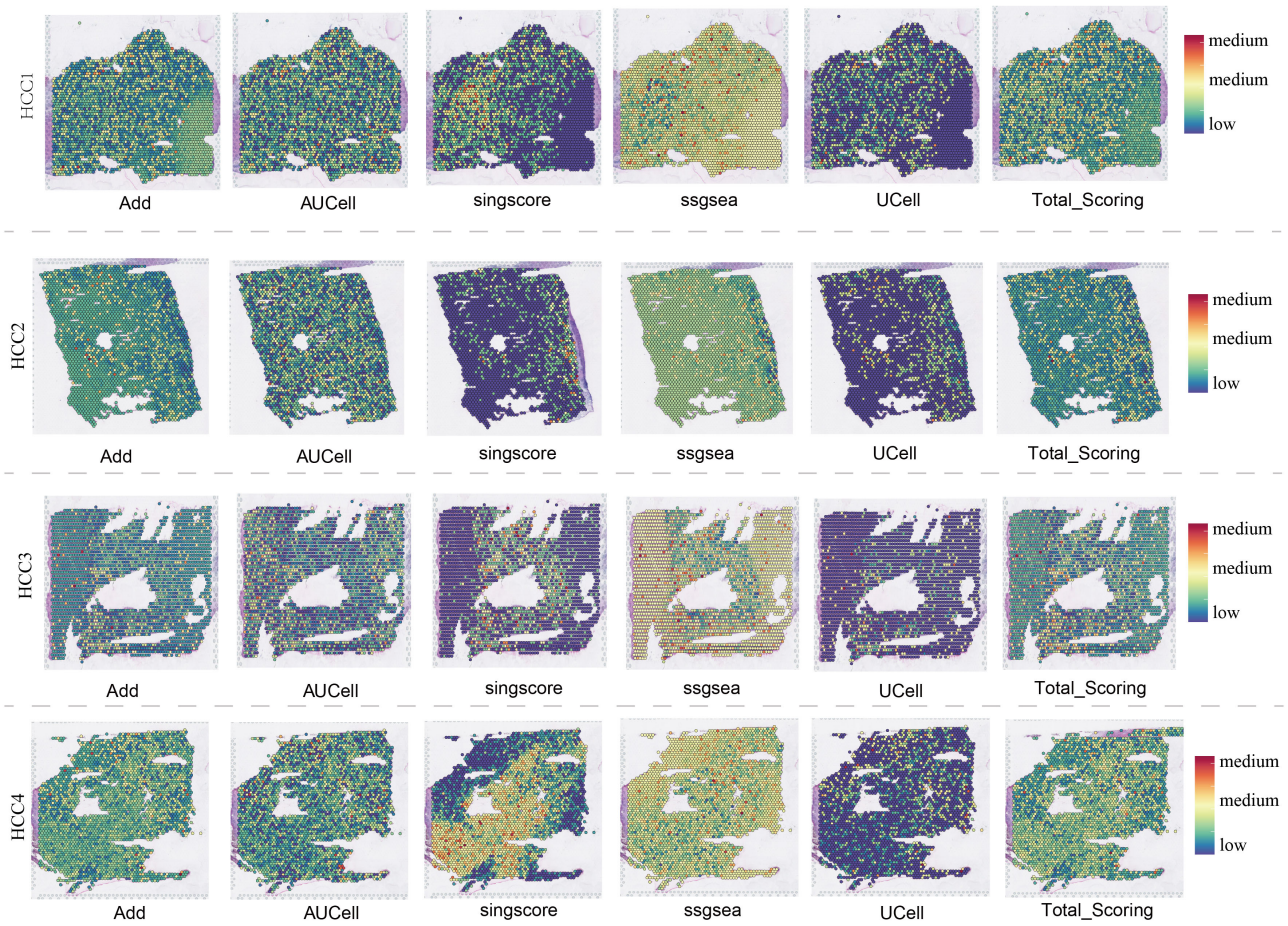
Spatial transcriptomics overview of 4 HCC samples.

The spatial distributions of ADAM family signals in malignant and benign cells under six different algorithms, AUCell, UCell, Add, singscore, ssgsea, and scoring, were shown in **Supplementary Figure 5E**. Quantitative analysis revealed significant differences in ADAM family signals between malignant and benign cells, emphasizing the crucial role of the ADAM family in the pathophysiology of HCC (**Supplementary Figure 5F**). Spatial transcriptomics provided the first spatial resolution imaging map of ADAM family signals across seven liver cancer tissues (**Figure 4**; **Supplementary Figures 6A-C**).

### Genomics and transcriptomics analysis of ADAM family members in multiple human cancers

**Figure 5:**
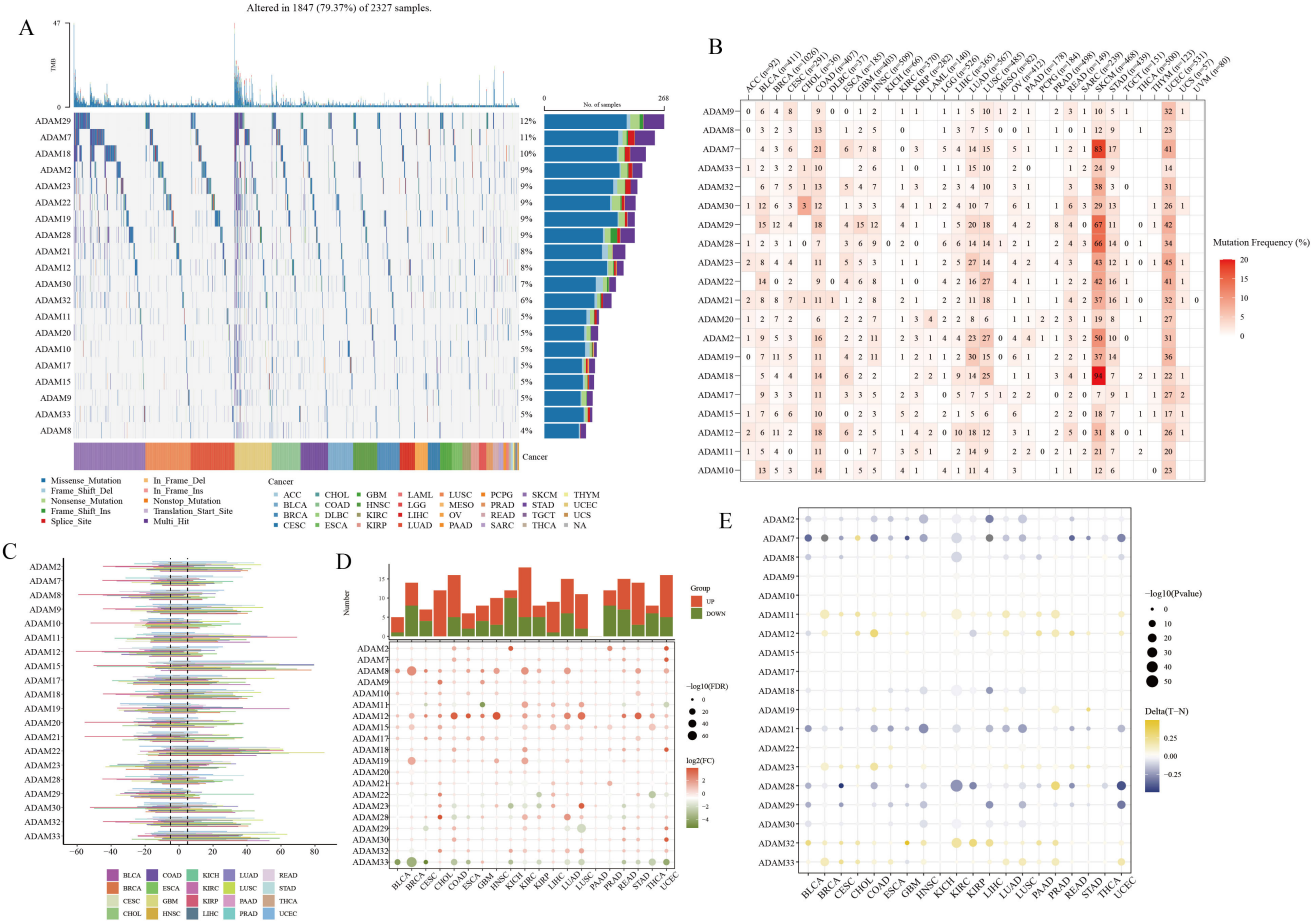
Pan-cancer overview of ADAM family members. **(A, B)** SNV characteristics of ADAM family members in multiple human cancers. **(C)** CNV characteristics of ADAM family members in multiple human cancers. **(D)** mRNA expression characteristics of ADAM family members in multiple human cancers. **(E)** Methylation characteristics of ADAM family members in multiple human cancers.

Pan-cancer SNV analysis revealed relatively high mutation frequencies in ADAM29, ADAM7, and ADAM18 (**Figure 5A**), with mutations predominantly observed in SKCM and UCEC (**Figure 5B**). Importantly, it’s worth noting that there are hardly any mutations of ADAM in HCC (**Figure 5B**). In addition to SNV analysis, CNV analysis were also conducted, with the results showing that ADAM15 and ADAM22 had relatively high CNV amplication (**Figure 5C**). The results of the DEGs analysis indicated a noteworthy pattern (**Figure 5D**). Specifically, ADAM8 and ADAM12 consistently exhibited a significant upregulation trend in most cancer types. In contrast, ADAM33 consistently displayed a significant downregulation trend across various cancers. Lastly, the methylation analysis revealed that ADAM11, ADAM32, and ADAM33 exhibited higher levels of methylation in tumor tissues compared to normal tissues. Conversely, ADAM7 and ADAM21 displayed lower levels of methylation in tumor tissues compared to normal tissues (**Figure 5E**).

### Molecular classifier of HCC based on ADAM family signals

**Figure 6:**
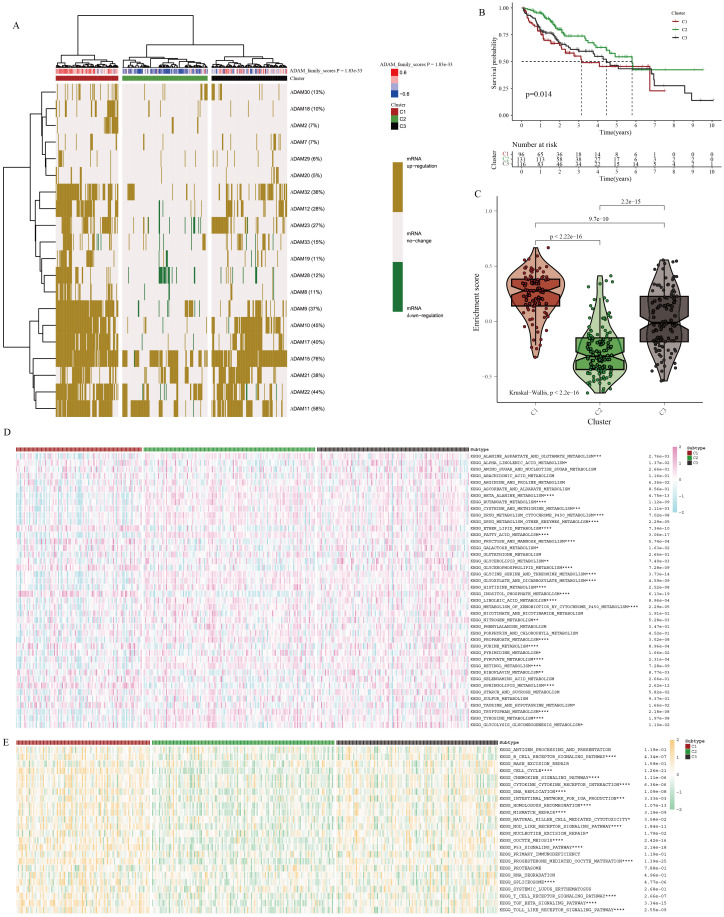
Identification of molecular characteristics of ADAM signals in HCC. **(A)** Cluster analysis for HCC patients based on ADAM signals. **(B)** Survival analysis of three clusters. **(C)** The violin plot displays the discrepancies in ADAM signals between three clusters. **(D)** The heatmap displays the discrepancies in metabolism traits between three clusters. **(E)** The heatmap displays the discrepancies in immune traits between three clusters. (*:p<0.05,**:p<0.01,***:p<0.001,****:p<0.0001; p value was calculated by kruskal.test).

In the TCGA-HCC cohort, a molecular classifier was developed based on the strength of ADAM family signals, successfully dividing TCGA-HCC patients into three subgroups (**Figure 6A**). HCC patients with varying ADAM signals displayed distinct clinical outcomes (**Figure 6B**). Among these subgroups, C1 exhibited the strongest ADAM family signals, followed by C3, and C2 showed the weakest signals (**Figure 6C**). Surprisingly, it was discovered that the stronger the ADAM family signals, the worse the prognosis for HCC patients, and vice versa. This further underscores the significant role of ADAM in the pathogenesis of HCC. Furthermore, it was observed that HCC patients with distinct ADAM signals were accompanied by varied metabolism and immune status (**Figures 6D, E**). As an illustration, individuals belonging to the C1 subgroup exhibited a marked reduction in beta-alanine metabolism and an increased activity in sphingolipid metabolism.

In addition to the immune pathways, we conducted an in-depth analysis of immune cell infiltration and immune checkpoint expression. Utilizing seven different immune deconvolution algorithms, we consistently observed that HCC patients with active ADAM signaling often exhibited a substantial influx of immune cells into the tumor microenvironment (**Supplementary Figure 7A**). Simultaneously, there was a notable abnormal activation of immune checkpoints (**Supplementary Figure 7B**). Excessive activation of immune checkpoints may favor the formation of an immune-suppressive microenvironment. Under such conditions, the body may trigger more compensatory responses, leading to the recruitment of an increasing number of immune cells into the tumor microenvironment, with the hope of controlling disease progression.

Considering the intricate regulatory interplay between ADAM signaling and the HCC immune microenvironment, we subsequently conducted more targeted immune-related analyses. Initially, utilizing the ssgsea algorithm and Spearman correlation test, we examined the associations between individual ADAM genes and classical immune-related pathways. The results highlighted that ADAM8, ADAM33, ADAM28, ADAM23, and ADAM19 were primarily positively correlated with immune pathways, while ADAM30 and ADAM11 exhibited predominantly negative correlations with immune pathways (**Supplementary Figure 8A**). Furthermore, the ADAM family signals displayed a predominantly positive correlation with immune pathways, particularly with pathways related to CCR, Treg, parainflammation, and macrophages (**Supplementary Figures 8B, C**).

### Machine learning-assisted development of a prognosis model based on ADAM family signals

**Figure 7:**
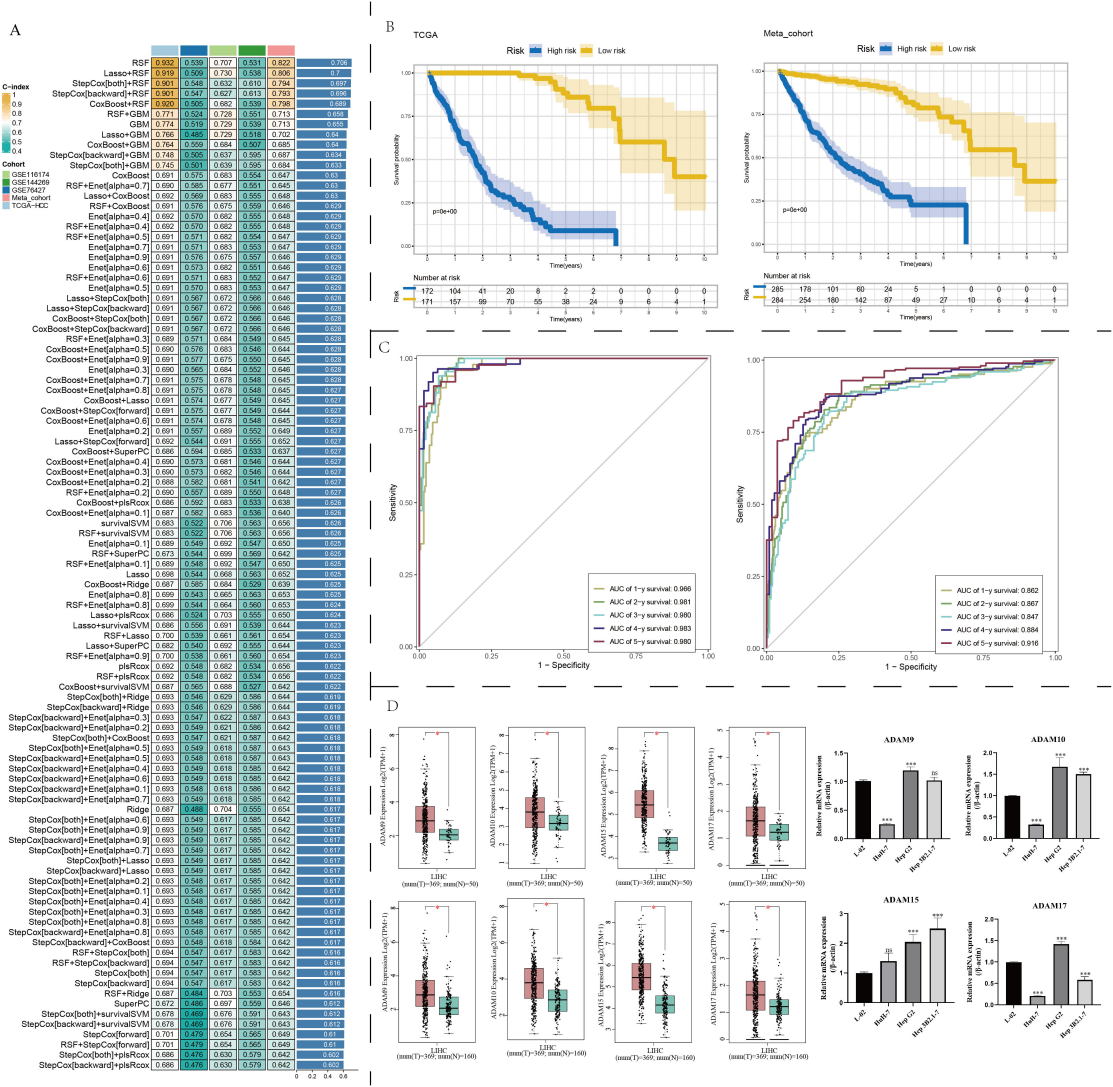
Machine learning determination of a robust prognostic signature associated with ADAM family members. **(A)** Find out the best prognostic model based on multiple machine learning algorithms. **(B)** Survival analysis of prognostic model. **(C)** ROC curves of prognostic model. **(D)** Expression traits of ADAM9, ADAM10, ADAM15, and ADAM17 based on GEPIA2 platform. qPCR experiments validated the expression of ADAM9, ADAM10, ADAM15, and ADAM17. (*:p<0.05, ***:p<0.001).

We utilized a diverse array of machine learning algorithms to search for the optimal prognosis model related to ADAM family signals. Given the limited number of ADAM family signal members, creating a precise and efficient molecular model based solely on ADAM family genes posed a significant challenge. Consequently, we conducted a comprehensive analysis by collecting and curating molecules that displayed significant interactions with ADAM family members, utilizing Spearman correlation analysis (|cor| > 0.3, p < 0.001). This effort yielded a total of 6,910 candidate molecules. Among these candidates, only 14 genes demonstrated prognostic significance across at least four liver cancer datasets. The Random Survival Forest (RSF) algorithm emerged as the most suitable approach for constructing the prognosis model, achieving the highest average C-index, approximately 0.706 (**Figure 7A**). Survival analysis results illustrate that this model effectively stratifies HCC patients into distinct risk groups, demonstrating its applicability in both the TCGA and Meta cohorts (**Figure 7B**). The ROC curve underscores its remarkable accuracy in prognostic assessment, with an AUC value of approximately 0.9 (**Figure 7C**). Lastly, we validated the expression levels of four key ADAM family genes. The results confirmed that ADAM9, ADAM10, ADAM15, and ADAM17 exhibited an upregulation trend in at least one liver cancer cell line, consistent with our earlier predictions (**Figure 7D**). Both our self-sequencing data and public single-cell data indicated that expression of ADAM9, ADAM10, ADAM15, and ADAM17 differed significantly between benign cells and malignant cells (**Figure 3F**; **Supplementary Figure 5G**).

### Clinical relevance analysis and expression validation of ADAM10

**Figure 8:**
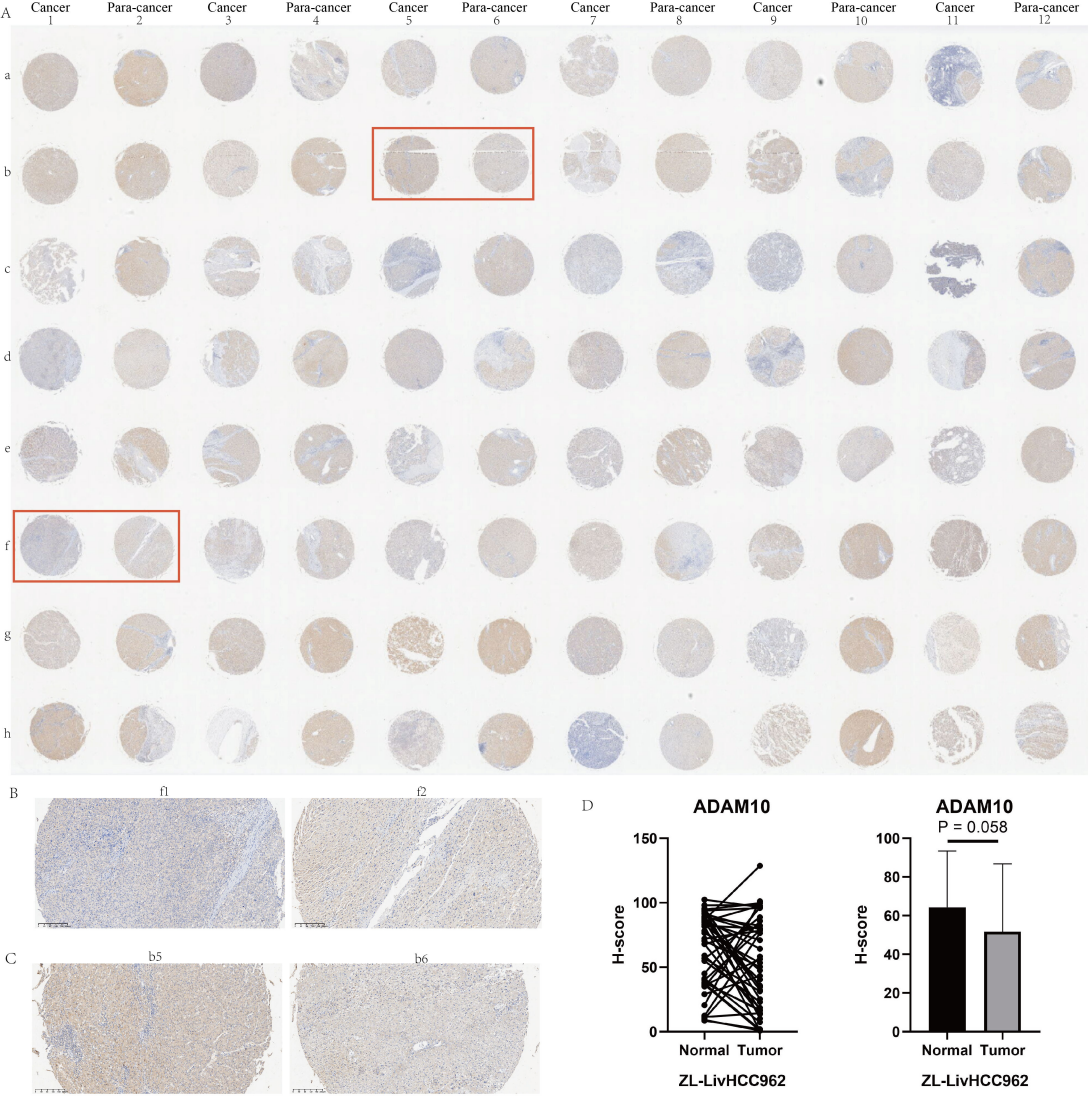
Immunohistochemical experiments on HCC tissue microarray of ADAM10. **(A)** Immunohistochemical profiles of 48 pairs of liver cancer patients. **(B)** Examples of low expression of ADAM10 in cancer tissue. **(C)** Examples of high expression of ADAM10 in cancer tissue. **(D)** Statistical quantitative analysis of ADAM10 protein levels by immunohistochemistry.

In addition to the TCGA dataset, nearly all liver cancer datasets indicate that the transcription levels of ADAM10 in liver cancer tissues were significantly higher than their corresponding adjacent tissues or normal liver tissue samples (**Supplementary Figure 9A**). The progression from hepatitis B infection to cirrhosis and eventually to liver cancer is a classic tumorigenic process, so we also investigated the expression profiles of ADAM10 in hepatitis B and cirrhosis patients. The results showed that liver cancer patients with concomitant HBV infection had higher levels of ADAM10 expression when compared to HBV-negative liver cancer patients (**Supplementary Figure 9B**). However, the presence of cirrhosis in liver cancer patients had no impact on the expression of ADAM10 (**Supplementary Figure 9B**). Furthermore, we also observed that higher ADAM10 expression was often found in liver cancer patients with the following characteristics: age equal to or less than 65 years (**Supplementary Figure 9C**), larger tumors (**Supplementary Figure 9D**), G3 and G4 stage patients (**Supplementary Figure 9E**), higher AFP levels (**Supplementary Figure 9F**), microsatellite lesions (**Supplementary Figure 9G**), TACE sensitivity (**Supplementary Figure 9H**), and sorafenib resistance (**Supplementary Figure 9I**). The immunohistochemical profiles of ADAM10 in 48 pairs of liver cancer tissues were shown in **Figure 8A**. At the protein level, most cancer tissue samples exhibited a downregulation trend (**Figure 8B**); however, there were also some patients’ cancer tissue samples that showed the opposite trend (**Figure 8C**). The immunohistochemical scores for these 48 pairs of liver cancer patients were displayed in **Figure 8D**, indicating a potential downregulation trend in the protein levels of ADAM10 in liver cancer tissues compared to adjacent tissues.

## Discussion

HCC is the most common type of primary liver cancer (34). Tumor invasion, metastasis, recurrence, and drug resistance have been identified as the main reasons for poor clinical outcomes in HCC patients. Therefore, finding new therapeutic targets for HCC has become an urgent priority. Members of the ADAM family genes are classified as classic transmembrane proteins and are widely recognized for their significant relevance to the prognosis of numerous chronic inflammatory diseases and cancers (35). Prior research (36) has documented a noteworthy upregulation of ADAM family members, particularly ADAM9, ADAM10, and ADAM17, in liver cancer, indicating a close link with tumor advancement. Our study corroborates these findings, as we identified abnormal overexpression of ADAM9, ADAM10, ADAM15, and ADAM17 in liver cancer tissues through both GEPIA website analysis and PCR experiments. Additionally, this trend was consistently observed in at least one liver cancer cell line.

This study employed a novel multi-omics integration approach to investigate the crucial role of ADAM signaling in HCC. Single-cell sequencing analysis revealed that ADAM signaling was predominantly activated in monocyte macrophages, endothelial cells, and hepatocytes, with significant differences observed in ADAM signaling between benign and malignant cells. Furthermore, this research provided the first spatially resolved characteristics of ADAM signaling. Cluster analysis based on ADAM signaling underscored once again that enhanced ADAM signaling could potentially lead to shortened patient survival, with tumor metabolic reprogramming and disruptions in the immune microenvironment being potential underlying causes of poor prognosis.

The mRNA levels of ADAM9 within HCC tissues serve as a prognostic indicator for reduced recurrence-free survival in cases of hepatitis B virus-related HCC (37). In cohorts of individuals with chronic hepatitis C, elevated sMICA levels, functioned as the ADAM9 substrate, following viral clearance were associated with HCC progression as a means to evade NK-mediated immune surveillance (38, 39). Combining agents that target ADAM9 activity with conventional multi-kinase inhibitors presents a promising future therapeutic approach to enhance the effectiveness of cancer management and treatment.

In addition to ADAM9, we have identified and validated the elevated expression of ADAM15 and ADAM17 in HCC. These findings are consistent with prior literature, which has reported that overexpression of ADAM15 is linked to a dismal prognosis in HCC and serves as an independent prognostic risk factor. Notably, the downregulation of ADAM15 promotes apoptosis in liver cancer cells and hampers tumor cell proliferation, migration, and invasion (40). The ADAM17 mRNA expression levels displayed variability across distinct pathological subtypes of HCC (41). ADAM17 enhances cell migration and invasion in hepatocellular carcinoma by modulating the integrin β1 pathway (42). In hepatocellular carcinoma cells, ADAM17 plays a role in hypoxia-induced drug resistance by activating the EGFR/PI3K/Akt pathway (43). A novel inhibitor of ADAM-17, ZLDI-8, suppresses hepatocellular carcinoma metastasis both *in vitro* and *in vivo* by reversing the process of epithelial-mesenchymal transition (19). In addition, ZLDI-8 augments the therapeutic impact of Sorafenib on hepatocellular carcinoma cells both *in vitro* and *in vivo* (18).

According to the research reported, the expression level of ADAM10 is elevated in pan-cancer, including lung cancer, pancreatic cancer, colon cancer, and breast cancer and so on (44–46). ADAM10 has been observed to be excessively expressed in HCC tissues (47), significantly correlating with tumor progression and reduced survival rates. In a cohort of 333 HCC patients, those carrying the ADAM10 rs514049 (AC + CC) variant were found to have a heightened susceptibility to develop lymph node metastasis, while individuals with the ADAM10 rs653765 variant were more prone to developing distant metastasis (47). After knocking down ADAM10, the proliferation, invasion, and migration of the HepG2 liver cancer cell line were significantly inhibited. Additionally, *in vivo* experiments confirmed the inhibition of tumor growth (10). This is consistent with the trends observed in our study, where we discovered and further confirmed the abnormally high expression of ADAM10. Furthermore, our analysis revealed a clear association between the levels of ADAM10 expression and tumor size as well as histological grade. Larger tumors and higher grade were often accompanied by elevated levels of ADAM10 expression. Zhang and his colleagues’ research have shown that the inhibition of ADAM10 can augment the therapeutic efficacy of sorafenib in treating HCC. Our analysis further corroborates these findings by indicating reduced ADAM10 expression in the sorafenib-responsive group. In summary, the combination of ADAM10 targeting and sorafenib for cancer treatment appears to be a promising and viable approach, necessitating future clinical trials for validation.

As for the transcript and protein expression trend of ADAM10 in patients with HCC, the results were same as previous researches absolutely based on the datasets from the TCGA, GEO, UALCAN and HPA. All these analysis were showing the science, accuracy and stability of the results. What’s more, the importance role of ADAM10 in HCC was depicted. And we found the high ADAM10 expression level is associated with prognostic risk factors, including the HBV status, tissue grading and satellite lesion. These might be the reason why the high expression level of ADAM10 contributed to the poor prognosis for HCC. And the expression level of ADAM10 is closely related to the pathological grade of HCC. We could predict the prognosis and choose suitable treatment therapy for HCC patients in future. It’s worth noting that our study revealed that the patients with high expression of ADAM10 are more sensitive in TACE therapy and more than more insensitive in sorafenib therapy than that for patients with low expression of ADAM10 for the first time. These could help clinical doctors in choosing suitable treatment methods, which can significantly enhance patient prognosis and hold great value and importance. Given the important role that ADAM10 plays in both pan-cancer and HCC, we further analyzed the prognostic value of ADAM10 in both pan-cancer and HCC by single-factor Cox regression analysis and log-rank test. As we could see, the ADAM10 plays well or poor role in the prognosis of different human tumors. Therefore, further studies are needed to research the specific role of ADAM10 in each pan-cancer.

There are still some limitations in this study that need to be addressed. Although the molecular classifiers and prognostic models derived from the ADAM gene family have been repeatedly validated using multiple public datasets, extensive real-world data is still needed for validation before they can be implemented in clinical practice.

## Conclusions

Single-cell sequencing reveals that ADAM signaling is primarily activated in monocyte- macrophages, endothelial cells, and hepatocytes in HCC. Benign and malignant liver cells exhibit distinct ADAM signaling features. Spatial transcriptomics sequencing for the first time unveils the spatial activation patterns of ADAM signaling in the liver. Aberrant activation of the ADAM family signals is a significant prognostic indicator for poor outcomes in HCC patients.

## Data Availability

The original contributions presented in the study are included in the article/**Supplementary Material**, further inquiries can be directed to the corresponding author/s.

## Supplementary material

Supplementary Figure 1 Quality control of our self-generated single-cell data. **(A)** Association of nCount_RNA, nFeature_RNA, and percent.rb. **(B)** Traits of nCount_RNA, nFeature_RNA, prevent.mt, and percent.rb. **(C)** tSNE characteristics before harmony correction. **(D)** tSNE characteristics after harmony correction.

Supplementary Figure 2 Quality control of public single-cell sequencing data. **(A)** Association of nCount_RNA, nFeature_RNA, and percent.rb. **(B)** Traits of nCount_RNA, nFeature_RNA, prevent.mt, and percent.rb. **(C)** tSNE characteristics before harmony correction. **(D)** tSNE characteristics after harmony correction. **(E)** Selection of highly selection genes and PC numbers. **(F)** Cluster tree for selecting the optimal resolution.

Supplementary Figure 3 Single-cell overview of HCC based on public sequencing data. **(A)** Cell type annotation. **(B)** t-SNE dimensionality reduction. **(C)** UMAP dimensionality reduction.

Supplementary Figure 4 Single-cell distribution of ADAM family signals based on public sequencing data. **(A)** Bubble chart displays the ADAM signals for each type of cell. **(B)** Violin plot displays the ADAM signals based on six algorithms. **(C)** t-SNE dimensionality reduction displays the single-cell distribution of ADAM signals. Six algorithms used for assessing ADAM signals involve AUCell, UCell, Add, singscore, ssgsea, and Scoring.

Supplementary Figure 5 Copykat results and cell cycle analysis of public single-cell sequencing data. **(A)** t-SNE dimensionality reduction of copykat results. **(B)** t-SNE dimensionality reduction of cell cycle analysis. **(C)** The proportion of G1, S, and G2M in HCC. **(D)** The proportion of aneuploid and diploid in HCC. **(E)** Single-cell distribution of ADAM signals in aneuploid and diploid. **(F)** The violin plot displays the discrepancies in ADAM signals between aneuploid and diploid. **(G)** The violin plot displays the discrepancies in expression of ADAM9, ADAM10, ADAM15, and ADAM17 between aneuploid and diploid. (***:p<0.001,****:p<0.0001; p value was calculated by wilcox.test).

Supplementary Figure 6 Spatial transcriptomics overview of HCC5, HCC6, and HCC7. **(A)** Spatial transcriptomics characteristics of ADAM signals in HCC5. **(B)** Spatial transcriptomics characteristics of ADAM signals in HCC6. **(C)** Spatial transcriptomics characteristics of ADAM signals in HCC7. Notable, HE figures were downloaded from the public website (i.e. GEO). (*:p<0.05,**:p<0.01,***:p<0.001).

Supplementary Figure 7 Immune microenvironment analysis. **(A)** The discrepancies in the immunocyte infiltration among three clusters. **(B)** The discrepancies in the immune checkpoint expression among three clusters. (*:p<0.05,**:p<0.01,***:p<0.001; p value was calculated by wilcox.test).

Supplementary Figure 8 Association of ADAM family members with HCC immune. **(A)** The heatmap shows the correlation between ADAM family members and immune traits of HCC. **(B)** Association of ADAM signals with HCC immune. **(C)** Scatter plots show the correlation between ADAM signals with CCR, Treg, parainflammation, and macrophages. (*:p<0.05,^:p<0.01; p value was calculated by Spearman.test).

Supplementary Figure 9 Correlation between ADAM10 expression level and clinical features of HCC based on the GEO datasets. **(A)** The RNA expression level of ADAM10 between the normal and tumor tissue in the pan-cancer based on E_TABM_36, GSE144269, GSE14520, GSE54236 and TCGA LIHC datasets. **(B)** The association of ADAM10 expression level with HBV infection and liver cirrhosis in patients with HCC based on the E_TABM_36, GSE144269 and GSE14520 datasets. **(C-G)** The association of ADAM10 expression level with age (TCGA LIHC), tumor size (GSE14520 dataset), grades (TCGA LIHC), the expression level of AFP (GSE14520), satellite lesion (GSE10141) the in patients with HCC, respectively. **(H, I)** The association of ADAM10 expression level with the treatment therapy sensitivity of TACE (GSE104580) and sorafenib (GSE109211).
