# Supplementary figures and images for: Unraveling the role of ADAMs in clinical heterogeneity and the immune microenvironment of hepatocellular carcinoma: insights from single-cell, spatial transcriptomics, and bulk RNA sequencing
Source: Front Immunol. 2024 Sep 13;15:1461424. doi: 10.3389/fimmu.2024.1461424 (PMC11427318; doi:10.3389/fimmu.2024.1461424)

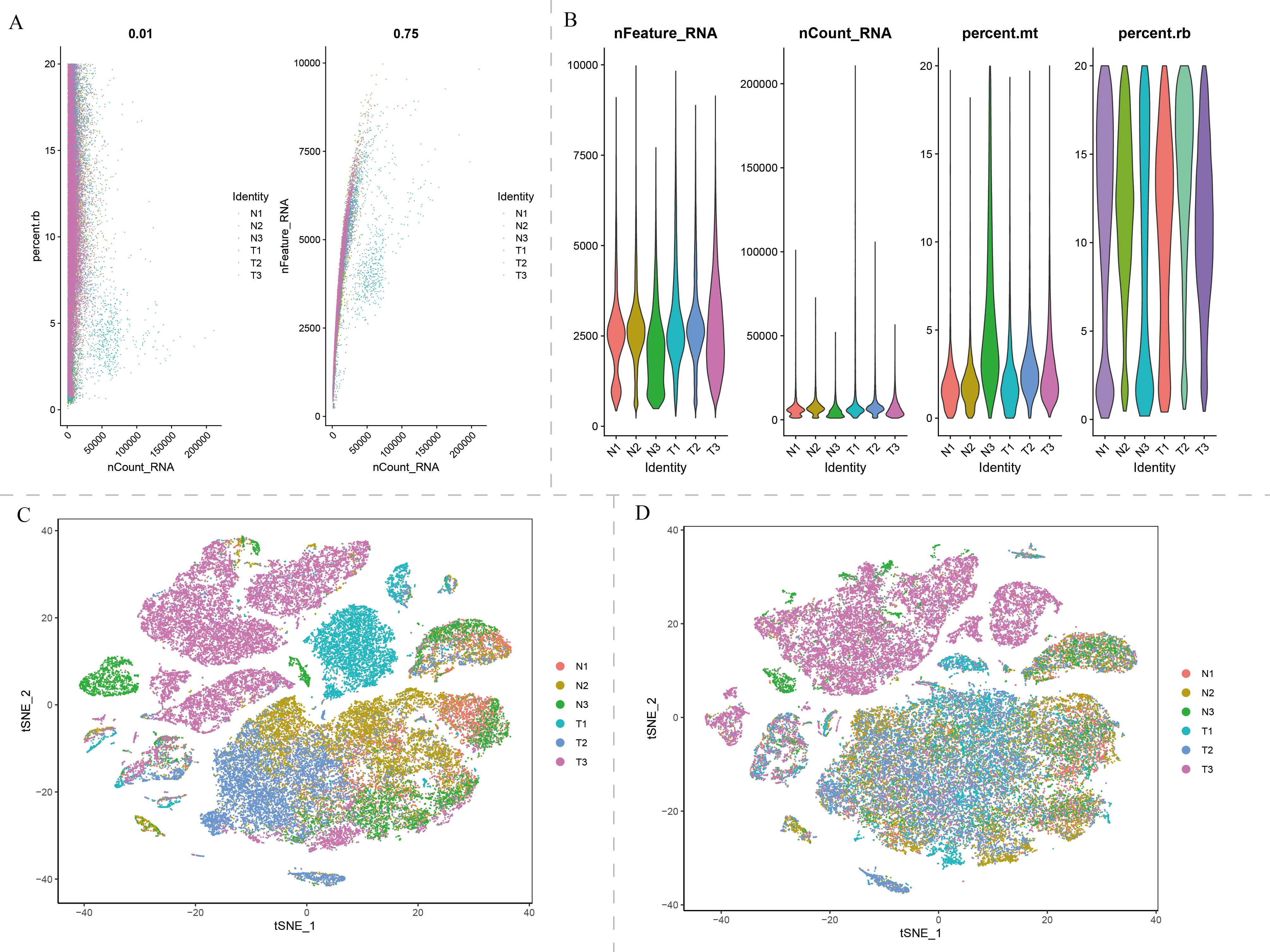

Supplement: Supplementary Figure 1 — Quality control of our self-generated single-cell data. (A) Association of nCount_RNA, nFeature_RNA, and percent.rb. (B) Traits of nCount_RNA, nFeature_RNA, prevent.mt, and percent.rb. (C) tSNE characteristics before harmony correction. (D) tSNE characteristics after harmony correction. [file Image1.jpeg]

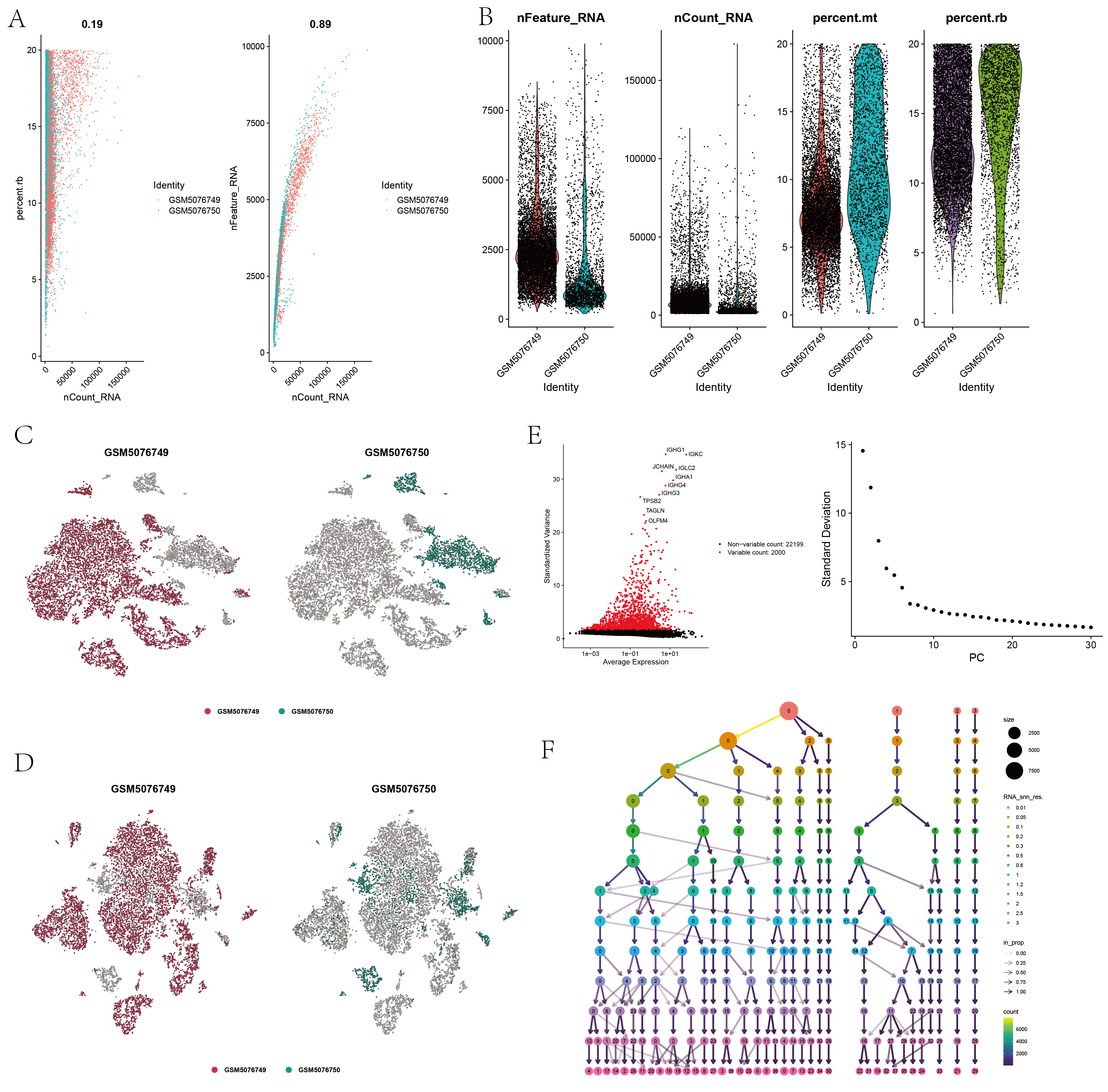

Supplement: Supplementary Figure 2 — Quality control of public single-cell sequencing data. (A) Association of nCount_RNA, nFeature_RNA, and percent.rb. (B) Traits of nCount_RNA, nFeature_RNA, prevent.mt, and percent.rb. (C) tSNE characteristics before harmony correction. (D) tSNE characteristics after harmony correction. (E) Selection of highly selection genes and PC numbers. (F) Cluster tree for selecting the optimal resolution. [file Image2.jpeg]

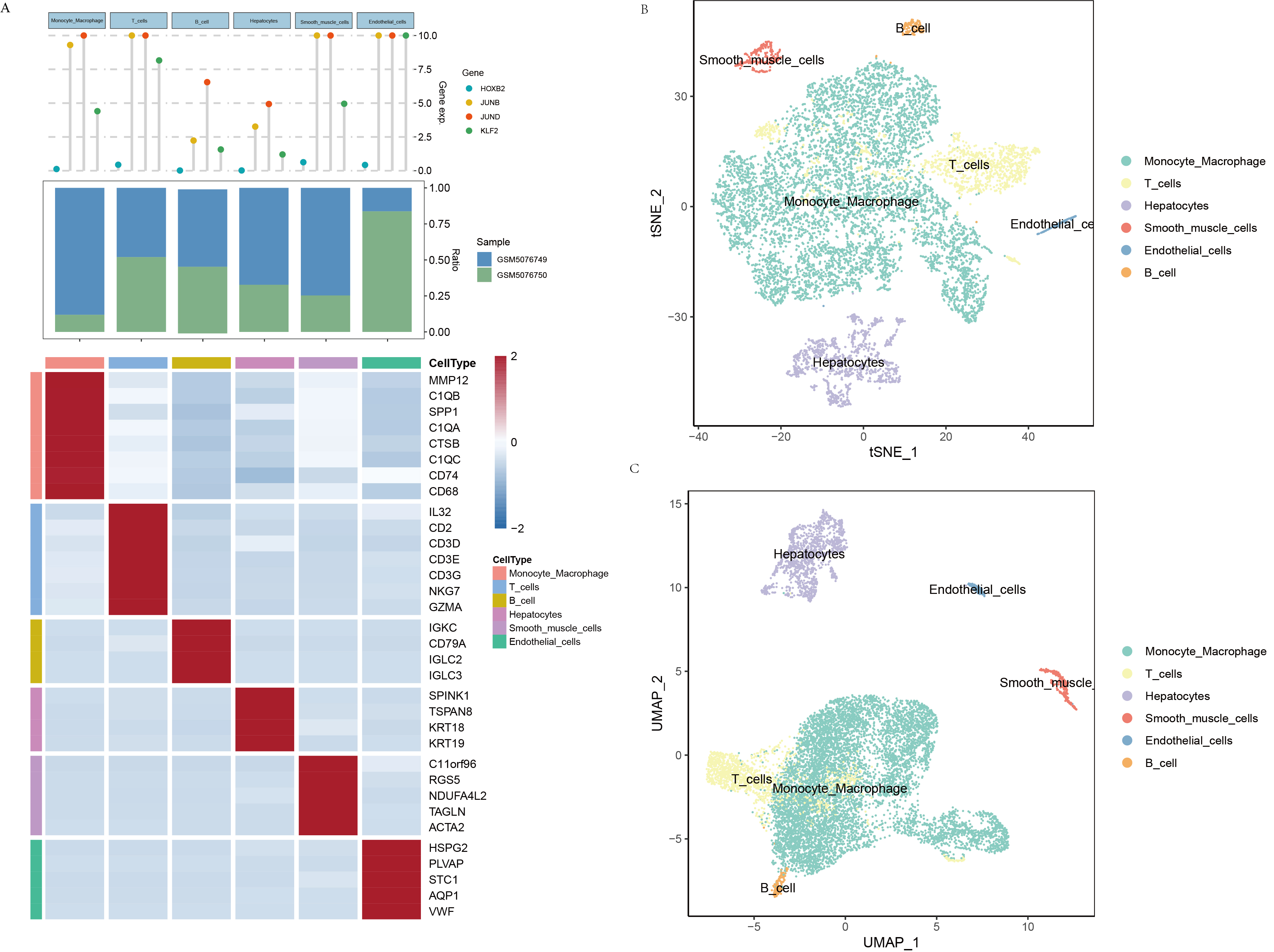

Supplement: Supplementary Figure 3 — Single-cell overview of HCC based on public sequencing data. (A) Cell type annotation. (B) t-SNE dimensionality reduction. (C) UMAP dimensionality reduction. [file Image3.jpeg]

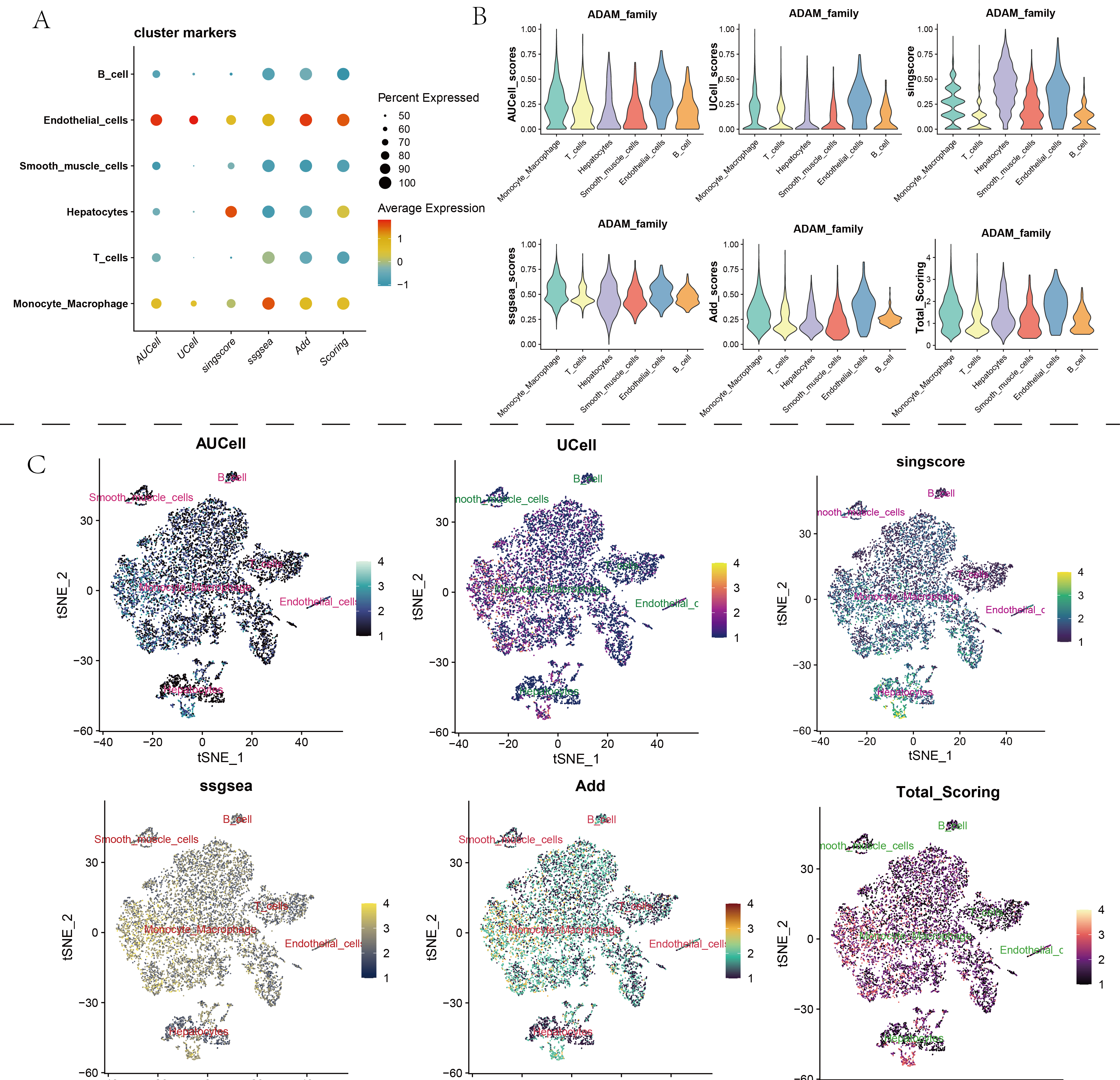

Supplement: Supplementary Figure 4 — Single-cell distribution of ADAM family signals based on public sequencing data. (A) Bubble chart displays the ADAM signals for each type of cell. (B) Violin plot displays the ADAM signals based on six algorithms. (C) t-SNE dimensionality reduction displays the single-cell distribution of ADAM signals. Six algorithms used for assessing ADAM signals involve AUCell, UCell, Add, singscore, ssgsea, and Scoring. [file Image4.jpeg]

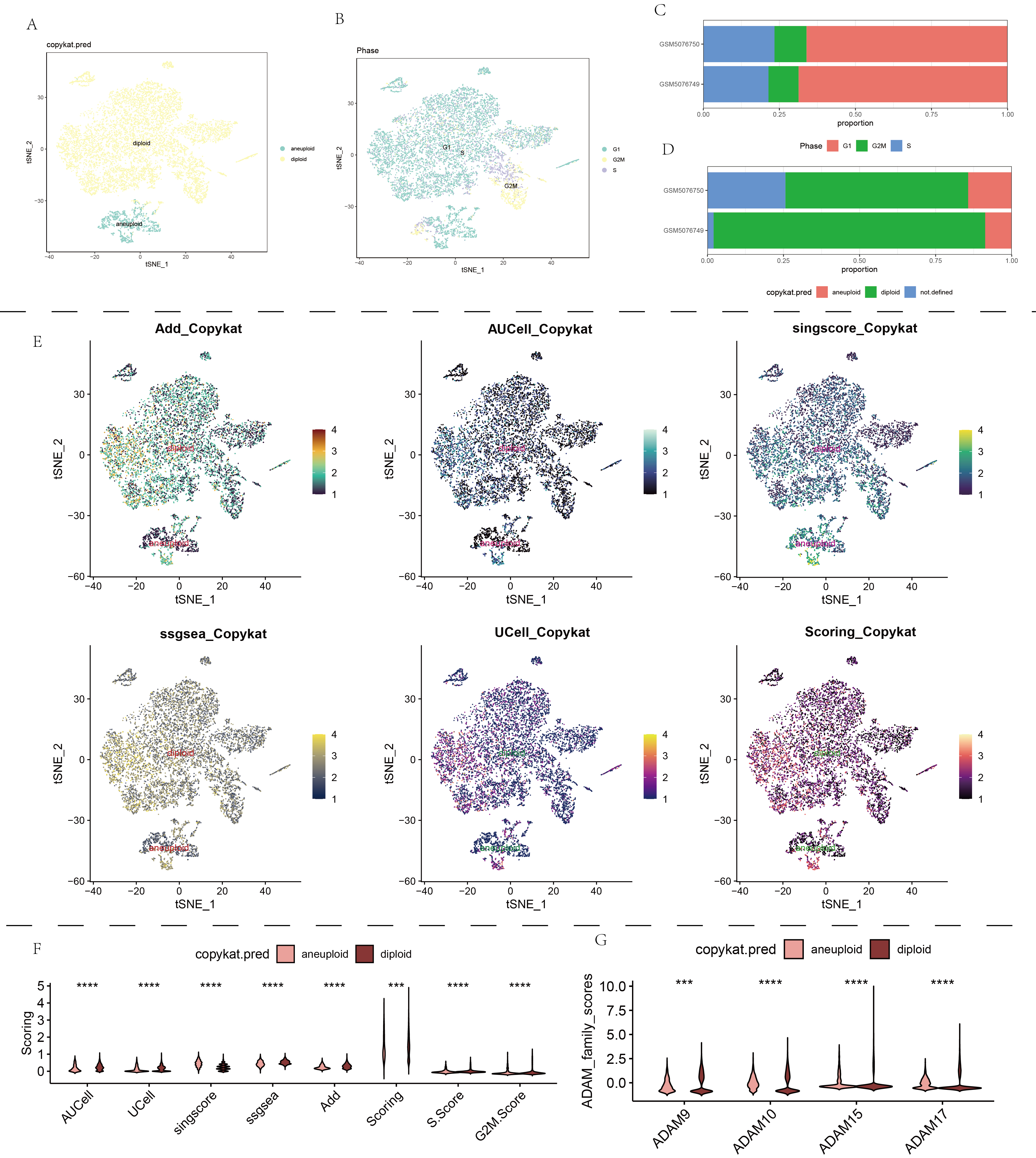

Supplement: Supplementary Figure 5 — Copykat results and cell cycle analysis of public single-cell sequencing data. (A) t-SNE dimensionality reduction of copykat results. (B) t-SNE dimensionality reduction of cell cycle analysis. (C) The proportion of G1, S, and G2M in HCC. (D) The proportion of aneuploid and diploid in HCC. (E) Single-cell distribution of ADAM signals in aneuploid and diploid. (F) The violin plot displays the discrepancies in ADAM signals between aneuploid and diploid. (G) The violin plot displays the discrepancies in expression of ADAM9, ADAM10, ADAM15, and ADAM17 between aneuploid and diploid. (***:p<0.001,****:p<0.0001; p value was calculated by wilcox.test). [file Image5.jpeg]

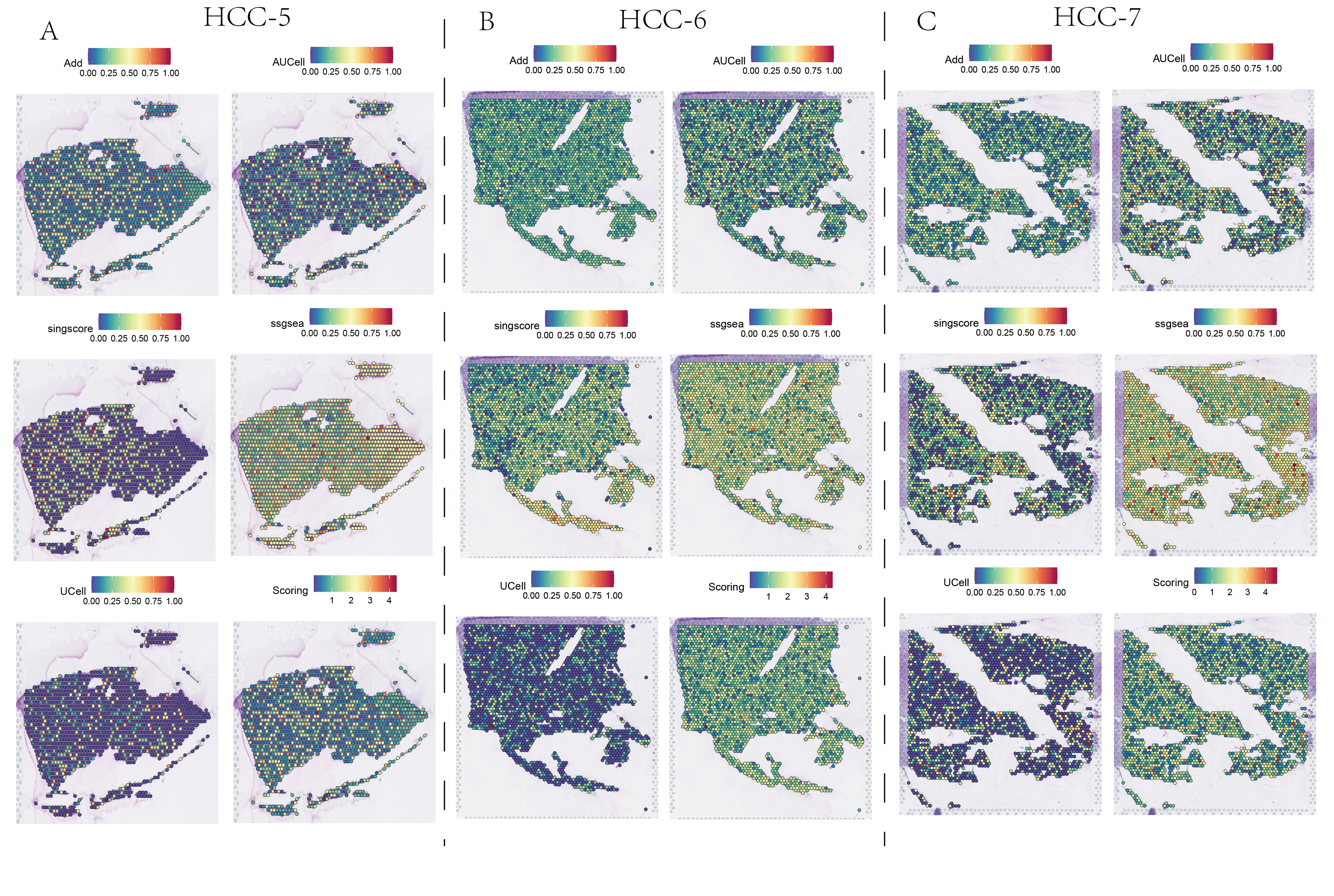

Supplement: Supplementary Figure 6 — Spatial transcriptomics overview of HCC5, HCC6, and HCC7. (A) Spatial transcriptomics characteristics of ADAM signals in HCC5. (B) Spatial transcriptomics characteristics of ADAM signals in HCC6. (C) Spatial transcriptomics characteristics of ADAM signals in HCC7. Notable, HE figures were downloaded from the public website (i.e. GEO). (*:p<0.05,**:p<0.01,***:p<0.001). [file Image6.jpeg]

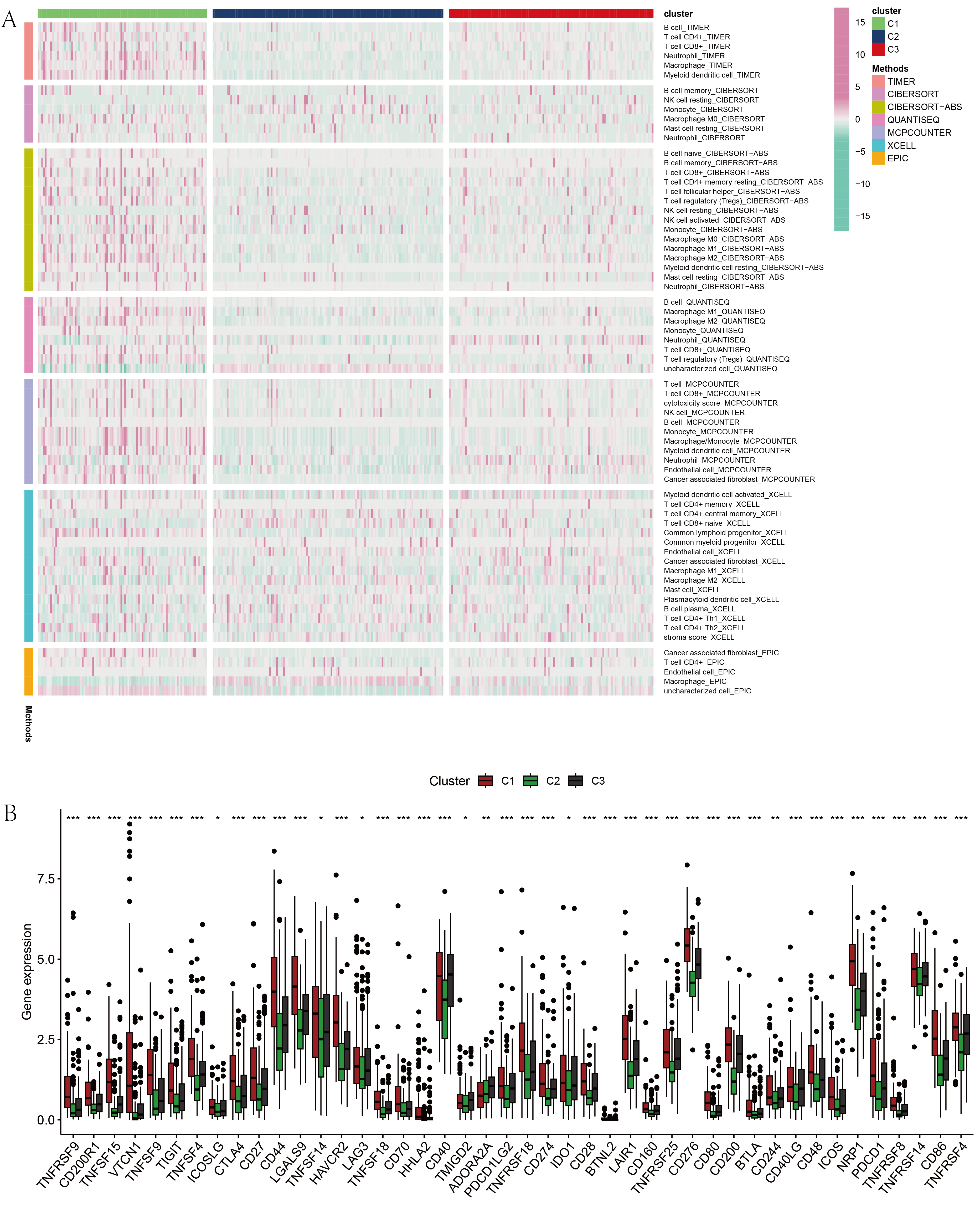

Supplement: Supplementary Figure 7 — Immune microenvironment analysis. (A) The discrepancies in the immunocyte infiltration among three clusters. (B) The discrepancies in the immune checkpoint expression among three clusters. (*:p<0.05,**:p<0.01,***:p<0.001; p value was calculated by wilcox.test). [file Image7.jpeg]

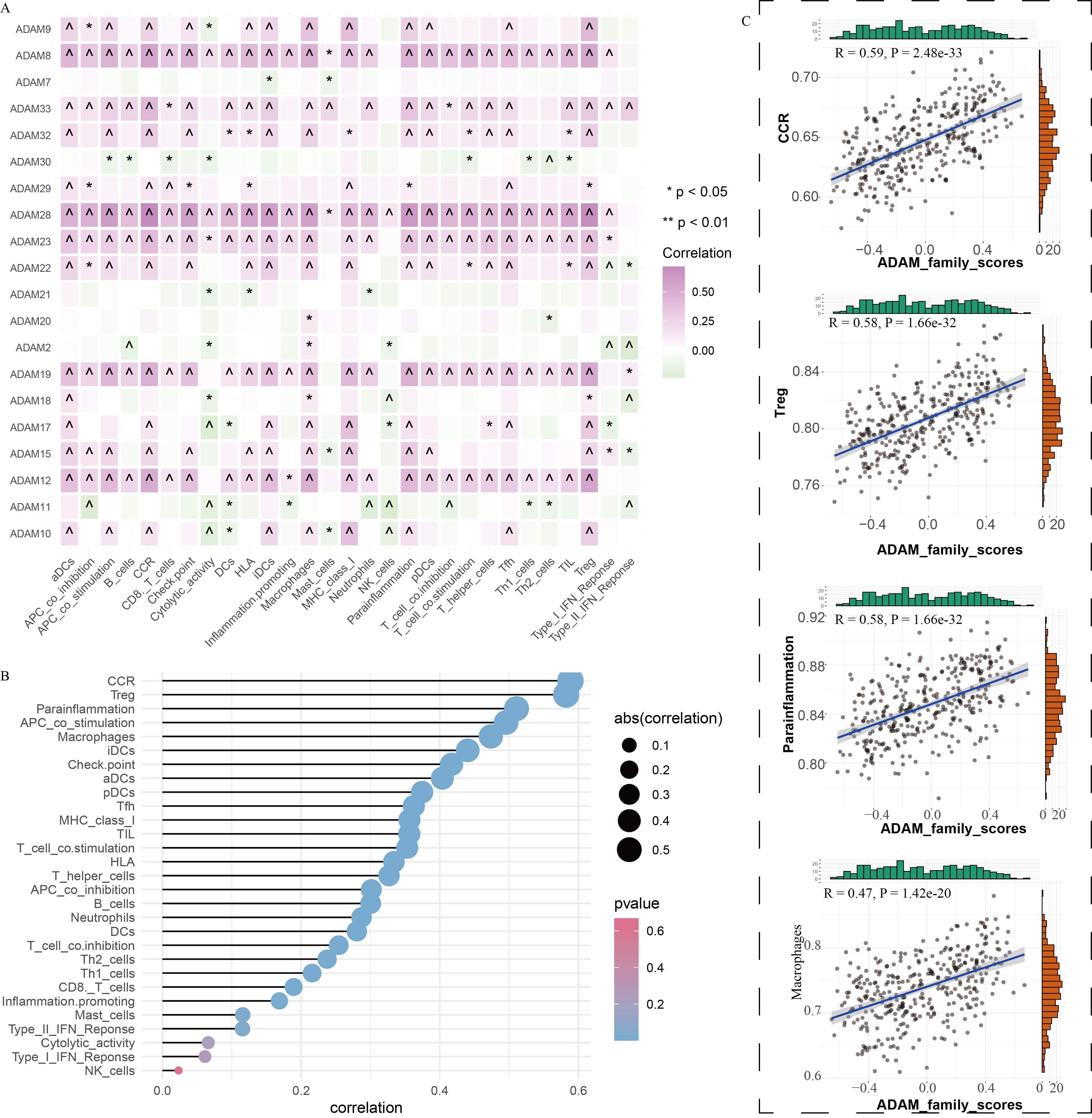

Supplement: Supplementary Figure 8 — Association of ADAM family members with HCC immune. (A) The heatmap shows the correlation between ADAM family members and immune traits of HCC. (B) Association of ADAM signals with HCC immune. (C) Scatter plots show the correlation between ADAM signals with CCR, Treg, parainflammation, and macrophages. (*:p<0.05,^:p<0.01; p value was calculated by Spearman.test). [file Image8.jpeg]

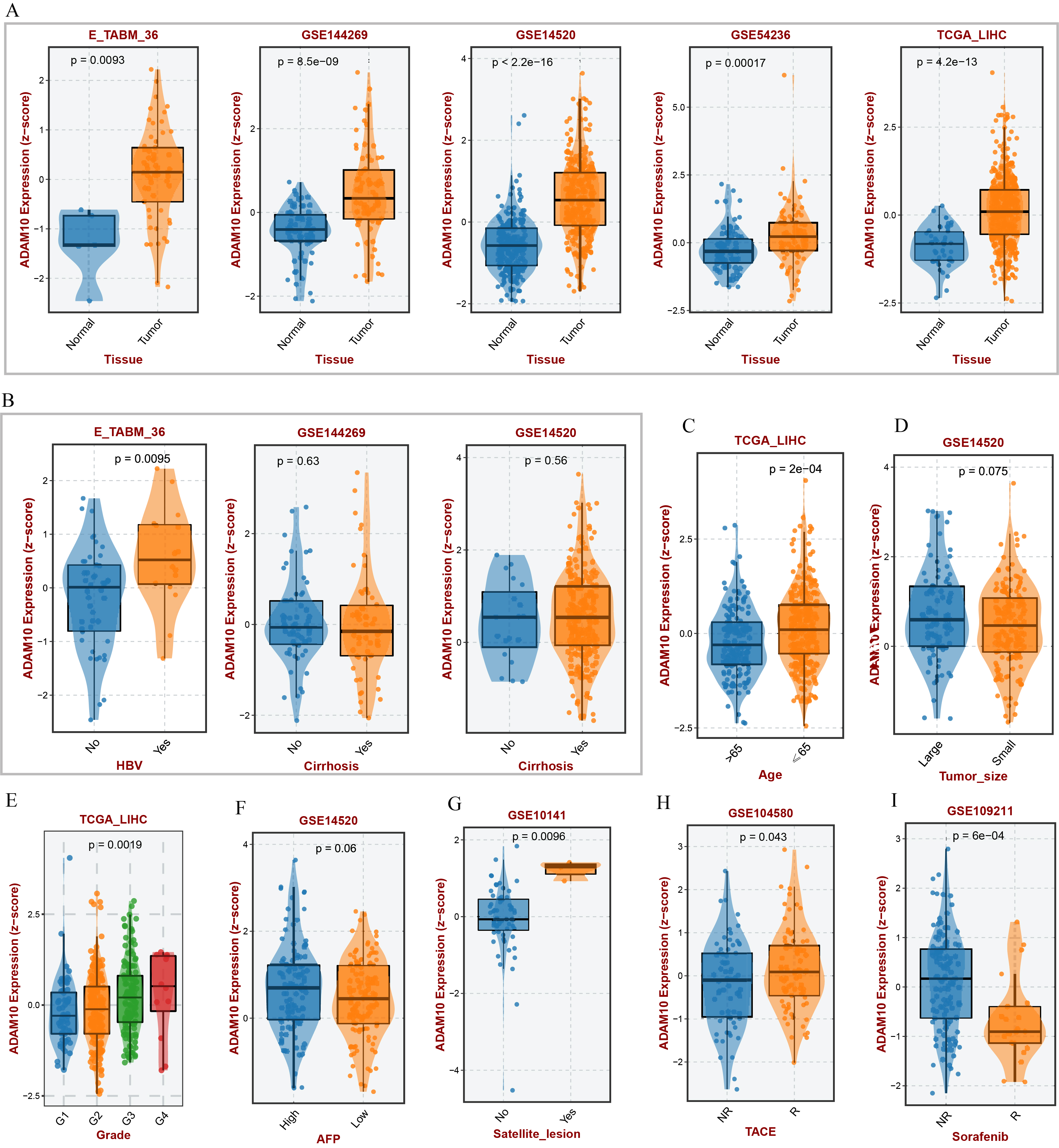

Supplement: Supplementary Figure 9 — Correlation between ADAM10 expression level and clinical features of HCC based on the GEO datasets. (A) The RNA expression level of ADAM10 between the normal and tumor tissue in the pan-cancer based on E_TABM_36, GSE144269, GSE14520, GSE54236 and TCGA LIHC datasets. (B) The association of ADAM10 expression level with HBV infection and liver cirrhosis in patients with HCC based on the E_TABM_36, GSE144269 and GSE14520 datasets. (C-G) The association of ADAM10 expression level with age (TCGA LIHC), tumor size (GSE14520 dataset), grades (TCGA LIHC), the expression level of AFP (GSE14520), satellite lesion (GSE10141) the in patients with HCC, respectively. (H, I) The association of ADAM10 expression level with the treatment therapy sensitivity of TACE (GSE104580) and sorafenib (GSE109211). [file Image9.jpeg]
